# In Silico Safety Assessment of *Bacillus* Isolated from Polish Bee Pollen and Bee Bread as Novel Probiotic Candidates

**DOI:** 10.3390/ijms25010666

**Published:** 2024-01-04

**Authors:** Ahmer Bin Hafeez, Karolina Pełka, Randy Worobo, Piotr Szweda

**Affiliations:** 1 Department of Pharmaceutical Technology and Biochemistry, Faculty of Chemistry, Gdańsk University of Technology, Ul. G. Narutowicza 11/12, 80-233 Gdańsk, Poland; ahmer.bin.hafeez@pg.edu.pl (A.B.H.); karolina.pelka@pg.edu.pl (K.P.); 2 Department of Food Science, Cornell University, Ithaca, NY 14853, USA; rww8@cornell.edu

**Keywords:** bee pollen, bee bread, *Bacillus* spp., probiotics, safety evaluation, pan-genome

## Abstract

*Bacillus* species isolated from Polish bee pollen (BP) and bee bread (BB) were characterized for in silico probiotic and safety attributes. A probiogenomics approach was used, and in-depth genomic analysis was performed using a wide array of bioinformatics tools to investigate the presence of virulence and antibiotic resistance properties, mobile genetic elements, and secondary metabolites. Functional annotation and Carbohydrate-Active enZYmes (CAZYme) profiling revealed the presence of genes and a repertoire of probiotics properties promoting enzymes. The isolates BB10.1, BP20.15 (isolated from bee bread), and PY2.3 (isolated from bee pollen) genome mining revealed the presence of several genes encoding acid, heat, cold, and other stress tolerance mechanisms, adhesion proteins required to survive and colonize harsh gastrointestinal environments, enzymes involved in the metabolism of dietary molecules, antioxidant activity, and genes associated with the synthesis of vitamins. In addition, genes responsible for the production of biogenic amines (BAs) and D-/L-lactate, hemolytic activity, and other toxic compounds were also analyzed. Pan-genome analyses were performed with 180 *Bacillus subtilis* and 204 *Bacillus velezensis* genomes to mine for any novel genes present in the genomes of our isolates. Moreover, all three isolates also consisted of gene clusters encoding secondary metabolites.

## 1. Introduction

Honeybees are social insects that live in large communities [1]. These honeybees in their hives produce and store several products that are beneficial for human health [2]. Indubitably, honey is the most prominent and widely valued product of the honeybee [2]. Ancient civilizations have relied on these products for centuries to treat various ailments, including wound healing, gut diseases, gastric ulcers, coughs, and sore throats in traditional medicine since ancient times [3]. A comprehensive review discussing the health effects of these bee products can be found elsewhere [2,4]. However, other products, including bee pollen (BP), propolis, bee bread (BB), royal jelly (RJ), and beeswax (BW), have also attracted the interest of the scientific community worldwide over the last few years [2]. Numerous studies have found beneficial effects of these natural products on human health, highlighting their potential use as active pharmaceutical ingredients [5,6,7,8,9,10,11,12]. 

Raw honey serves as a reservoir for several microbial species, including molds, yeasts, and spore-forming bacteria [13], with *Bacillus* and *Lactobacillus* species predominantly found [14,15,16]. On the other hand, bee pollen (BP) and, particularly, bee bread (BB) are less known and less popular among consumers. Recent research has uncovered the impressive health benefits of these bee products, which include antimicrobial, antioxidant, anti-radiation, anti-inflammatory, anti-tumor, hepatoprotective, and chemoprotective activities [17,18,19,20,21].

BB is fermented flower pollen collected by bees and stored in honeycombs, where it undergoes a transformation process facilitated by enzymes from bee glands and gut microbiota, particularly lactic acid bacteria (LAB), *Bacillus* sp., and yeasts [18,22,23]. Rich in carbohydrates, protein, lipids, and various micronutrients such as minerals, vitamins, phenolic compounds, and essential amino acids, BB serves as a food source, primarily for young bees and larvae within the hive [2]. The fermentation process acts as a natural preservative, preventing spoilage and the growth of pathogenic bacteria, ensuring the safety of both bees and humans who consume this product [17]. BP and BB exert several health benefits. For example, BP and BB ameliorate blood sugar, amend diabetic testicular–pituitary system dysfunction, prevent obesity, combat liver disorders, have cardio-protective effects, lower uric acid, etc. [24,25,26,27]. Several bacterial and yeast species have been isolated from BP and BB [28,29]. However, limited efforts have been made to explore the potential of isolates as probiotics.

Probiotics are live microorganisms that, when consumed in adequate amounts, provide health benefits to the host [30]. Probiotic microorganisms have been reported to improve gastrointestinal health, facilitate vitamin production, and make these essential compounds available to their host. Their beneficial impact extends to several health disorders, encompassing improvements in blood pressure, the sustained maintenance of blood cholesterol profiles, and positive associations with conditions like allergies, inflammatory bowel disease, bacterial vaginosis, dental caries, etc. [31]. Honey serves as a rich source of microorganisms, including fungi, lactic acid bacteria, and *Bacillus*, some of which exhibit probiotic properties [32,33,34,35]. Recent studies have identified bacteria from bee bread as potential probiotics [28,36,37,38]. Furthermore, certain strains of *Bacillus* and *Lactobacillus* isolated from fermented foods have shown promising probiotic potential [39]. *Bacillus* spp. are Gram-positive and spore-forming bacteria commonly found in soil and plants, have the advantage of being highly stable in acidic environments, and can thrive in various food matrices [40,41,42,43,44]. In our earlier study, we assessed ten bacterial isolates obtained from bee bread (BB) and bee pollen (BP) to determine their potential as probiotics. Following extensive wet lab experiments, only three isolates met the established probiotic criteria. Subsequently, we conducted whole-genome sequencing on these three strains, identifying them taxonomically as members of *Bacillus* [28]. In this study, our primary focus was to conduct a comprehensive probiogenomic analysis on three distinct *Bacillus* strains—BB10.1 and BP20.15 derived from bee bread and PY2.3 from bee pollen. The main objective was to assess the safety profile of these strains by investigating the presence of virulence genes, toxins, and genes responsible for conferring probiotic characteristics. 

## 2. Results

### 2.1. Genome Assessment and Synteny

**Figure 1:**
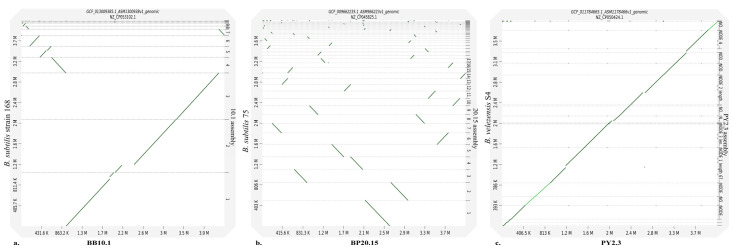
Synteny analysis of (**a**) BB10.1, (**b**) BP20.15, and (**c**) PY2.3. Pairwise genome alignment for the selected strains is shown in the dot plots generated by D-GENIES tool.

BUSCO analysis revealed a 99.6%, 99.5%, and 100% completeness of the genome for isolates BB10.1, BP20.15, and PY2.3, respectively. The percentages represent the completeness of our genome assemblies, indicating the proportion of conserved genes in a benchmark dataset. Higher percentages indicate better genome completeness. A total of 99.6%, 99.3%, and 99.7% of genes were recognized as single-copy BUSCO for BB10.1, BP20.15, and PY2.3, respectively. The high percentages indicate that a significant portion of genes are present as single-copy orthologs. Fragmented BUSCOs represent genes that are partially present or fragmented in our assemblies. A total of 0.2% and 0.4% of genes were present as fragmented copies for BB10.1 and BP20.15, respectively, while 0% of genes were missing for all three strains. A missing BUSCO indicates that a gene is absent from the assembly. The fact that there are no missing BUSCOs in our results suggests that our assemblies contain a comprehensive set of genes from the benchmark dataset (Table 1), which also provides confidence in our data for exploring the probiotic potential of these *Bacillus* strains. Furthermore, D-GENIES analyzed the genome synteny by pairwise genome alignment of the isolate with their closely related strains: BB10.1 with *B. subtilis* strain 168, BP20.15 with *B. subtilis* 75, and PY2.3 with *B. velezensis* S4. The dot plots revealed a few genomic region inversions between reference *B. subtilis* strain 168 and BB10.1, while several were observed between *B. subtilis* 75 and BP20.15 (Figure 1a,b). For PY2.3 and *B. velezensis* S4, minor variations were observed (Figure 1c). 

### 2.2. Pan-Genome Analysis

**Figure 2:**
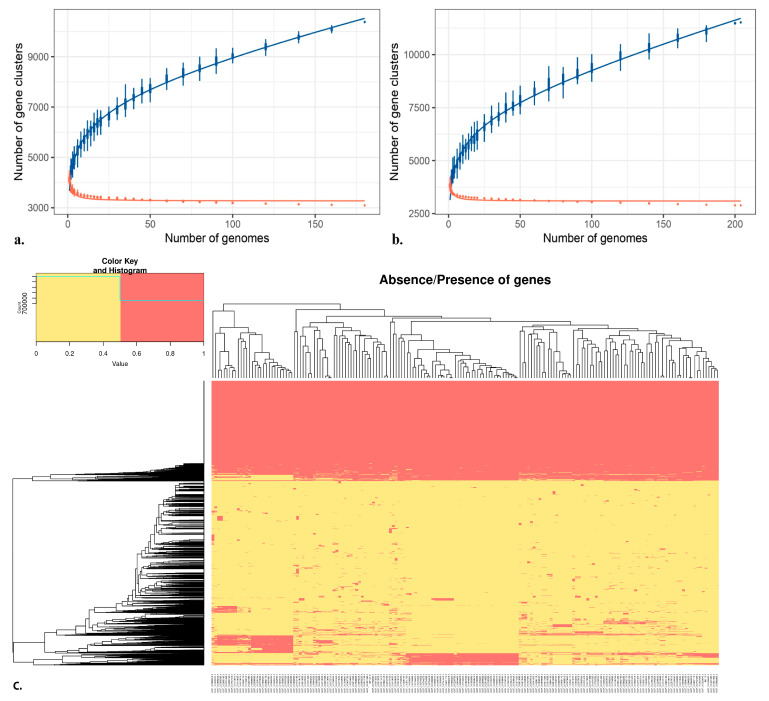
Representation of (**a**) BB10.1 and BP20.15 pan-genome and (**b**) PY2.3 gene content (extrapolated median-based line) according to how the pan-genome varies as genomes are added in random order to the analysis. The blue line represents unique genes; the red line represents new genes. The heatmap (**c**) represents the presence and absence of particular genes in BB10.1 and BP20.15 pan-genome. The *X*-axis denotes the particular species name. The yellow color indicates gene presence; the red color indicates gene absence. The *Y*-axis represents individual gene clustering, while the topmost axis represents genomes clustering (high-quality figure in Supplementary File).

*B. subtilis* pan-genomic analysis analyzed 752,925 genes, 10,810 total orthologous groups, and 2664 total unique genes, while the count for average core genes was 3499, the average gene count was 4207, and the average unique gene count was 15 (Supplementary Figure S1). Similarly, pan-genomic analysis of *B. velezensis* analyzed 790,409 genes, 11,759 total orthologous groups, and 3831 total unique genes, while the count for average core genes was 3346, the average gene count was 3821.5, and the average unique gene count was 10 (Supplementary Figure S2). Nevertheless, we observed that the number of new genes is progressively decreasing, proportionally to the number of genomes included in the *B. subtilis* (Figure 2a) and *B. velezensis* analyses (Figure 2b). The heatmap (Figure 2c) represents the absence and presence of genes in the BB10.1 and BP20.15 pan-genomes, whereas the gene presence/absence heatmap for PY2.3 is presented in Supplementary Figure S3. The phylogenetic inferences based on the whole genome variation of isolates BB10.1, BP20.15, and PY2.3 are presented in Supplementary Figure S4a and S4b, respectively.

#### Functional Annotation and Carbohydrate-Active Enzyme (CAZyme) Profiling

The KEGG functional annotation by BlastKOALA annotated and categorized the genes into 22 different functional categories (Supplementary Figure S5). For isolate BB10.1, 2455 entries (60.0%) were annotated out of 4092 and mostly related to protein families: genetic information processing (13.07%), carbohydrate metabolism (10.79%), protein families: signaling and cellular processes (10.54%), environmental information processing (9.04%), genetic information processing (7.37%), amino acid metabolism (5.45%), nucleotide metabolism (3.09%), metabolism of cofactors and vitamins (4.76%), and others. Similarly, 2437 entries (61.2%) out of 3980 were annotated for isolate BP20.15, and the most related were protein families: genetic information processing (13.00%), carbohydrate metabolism (10.91%), protein families: signaling and cellular processes (10.79%), environmental information processing (9.02%), genetic information processing (7.38%), amino acid metabolism (5.37%), nucleotide metabolism (3.03%), metabolism of cofactors and vitamins (4.84%), and others. For isolate PY2.3, 2335 entries were annotated out of 3729 (62.6%), mostly related to protein families: genetic information processing (13.19%), carbohydrate metabolism (10.32%), protein families: signaling and cellular processes (9.89%), environmental information processing (8.39%), genetic information processing (7.45%), amino acid metabolism (5.31%), nucleotide metabolism (3.08%), metabolism of cofactors and vitamins (5.01%), and others.

**Figure 3:**
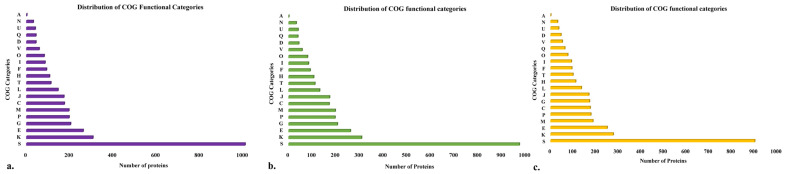
Distribution of cluster of orthologous group (COG) functional categories to the proteins of isolate (**a**) BB10.1; (**b**) BP20.15; (**c**) PY2.3. The *X*-axis denotes the number of proteins and *Y*-axis denotes COG categories. Each alphabet represents a unique COG category. A—RNA processing and modification, C—energy production and conversion, D—cell cycle control, cell division, and chromosome partitioning; E—amino acid transport and metabolism; F—nucleotide transport and metabolism; G—carbohydrate transport and metabolism; H—coenzyme transport and metabolism; I—lipid transport and metabolism; J—translation, ribosomal structure, and biogenesis; K—transcription; L—replication, recombination, and repair; M—cell wall/membrane/envelope biogenesis; N—cell motility; O—post-translational modification, protein turnover, chaperones; P—inorganic ion transport and metabolism; Q—secondary metabolites biosynthesis, transport, and catabolism; S—function unknown; T—signal transduction mechanisms; U—intracellular trafficking, secretion, and vesicular transport; and V—defense mechanisms.

Clusters of orthologous groups (COGs) functional group analysis was performed using EggNOG Mapper v2. For the isolate BB10.1, 3843 genes out of 4092 (93%) were assigned to 20 clusters. The function unknown (S: 1009) category had the highest numbers, implicating the uniqueness and yet-to-explore potential of this strain. The remaining proteins were categorized under functional groups such as transcription (K: 307); amino acid transport and metabolism (E: 262); carbohydrate transport and metabolism (G: 204); inorganic ion transport and metabolism (P: 197); energy production and conversion (C: 175); translation, ribosomal structure, and biogenesis (J: 173); cell wall/membrane/envelope biogenesis (M: 196); replication, recombination, and repair (L: 146); coenzyme transport and metabolism (H: 107); nucleotide transport and metabolism (F: 93); signal transduction mechanisms (T: 113); post-translational modification, protein turnover, chaperones (O: 82); lipid transport and metabolism (I: 86); defense mechanisms (V: 59); chromosome partitioning (D: 44); cell cycle control, cell division, secondary metabolites biosynthesis, transport, and catabolism (Q: 44); cell motility (N: 32); intracellular trafficking, secretion, and vesicular transport (U: 41); and RNA processing and modification (A: 2) (Figure 3a). A total of 3747 entries out of 3980 (94%) for strain BP20.15 were classified into 20 different COG categories (Figure 3b) and, for PY2.3, 3559 out of 3729 entries (95%) were assigned to 20 different COG categories (Figure 3c).

**Figure 4:**
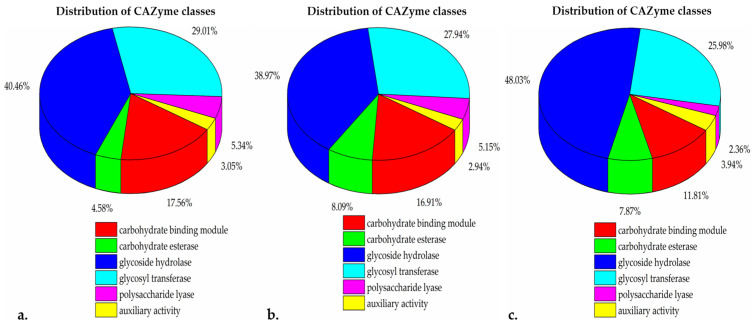
The percent distribution of CAZymes classes. (**a**). BB10.1, (**b**). BP20.15, (**c**). PY2.3. The color scheme at the bottom represents different CAZyme classes: red, CBM—carbohydrate-binding module; green, CE carbohydrate esterases; blue, GH—glycoside hydrolases; cyan, GT—glycosyl transferases; pink, PL—polysaccharide lyases; and yellow, AA—auxiliary activity.

PGAP [45] analysis resulted in 4092, 3980, and 3729 annotated protein sequences for BB10.1, BP20.15, and PY2.3, respectively. The meta server, dbCAN2, classified and annotated enzymes via all three tools. HMMER: dbCAN, DIAMOND: CAZy, and HMMER: dbCAN-sub. Enzymes classified by at least two tools were considered. From the isolate BB10.1 genome, 147 were identified by dbCAN as belonging to carbohydrate-active enzyme families. These included 35 conserved carbohydrate-active enzyme (CAZy) domains whose genes had signal peptides. These CAZy domains represented CAZy families, including 23 from the carbohydrate-binding module (CBM) family, 6 from the carbohydrate esterase (CE) family, 53 from the glycoside hydrolase (GH) family, 38 from the glycosyl transferase (GT) family, and 7 from the polysaccharide lyase (PL) family (Figure 4a). The isolate BP20.15 genome encoded 135 CAZy families, with 35 having conserved signal peptides. These CAZy domains include 23 from the CBM family, 11 from the CE family, 53 from the GH family, 38 from the GT family, 7 from the PL family, and 4 from the auxiliary activities (AAs) family (Figure 4b). On the other hand, the PY2.3 genome consists of 109 CAZy domains, and 31 of those 109 CAZy domains had conserved signal peptides. There was a total of 15 entries for the CBM family, 10 from the CE family, 61 from the GH family, 33 from the GT family, 3 from the PL family, and 1 from the AA family (Figure 4c). The signal-peptide-containing glycoside hydrolase family was the largest group of carbohydrate-active enzymes.

### 2.3. Probiotic- and Stress-Related Genes

Probiotic strains usually consist of an arsenal of genes encoding stress proteins in response to temperature, pH, bile, osmotic pressure, and oxidative stress that regulate their adaptability to the gastrointestinal (GI) tract (Journey of the Probiotic Bacteria: Survival of the Fittest). A literature-mining approach was used, and *Bacillus* and other related probiotic species’ published data were analyzed. The annotated genomes were analyzed to search for various probiotic-properties-related genes (stress resistance, bile salt hydrolase activity, adhesion ability, immunomodulatory activities) to determine the probiotic functions at genomic levels. Several genes encoding for stress-related proteins were identified in the genomes of isolate BB10.1, BP20.15, and PY2.3 as presented in (Table 2) and BLASTed in Uniprot.

The genomes of all the tree isolates were found to encode genes related to heat shock, including heat-shock-related regulators (*hrcA*, *ctsR*), molecular chaperones (*dnaK*, *dnaJ*, *grpE*, *groEL*, *groES*), and protease-encoding genes (*hslO*, *lon1*, *lon2*, *clpC*, *clpE*, *clpP*, *clpQ*, *clpX*, and *clpY*) [46,47,48,49,50,51]. These genes are considered to play a major role in intracellular protein aggregation and membrane stabilization to resist higher temperatures. Genes (*cspB*, *cspC*, *cspD*) coding for cold shock proteins [52,53] related to survival under low temperatures were also observed in the genomes of the isolates. The *CSP* family genes are synthesized by several *bacillus* strains to overcome the deleterious effect under cold stress and hence may provide resistance to the isolates. In addition, genes conferring resistance to low pH conditions were also observed. Out of the 14 genes observed, 9 genes encode a cluster of ATP synthase subunits A–I, which serve as a key regulator of cytoplasmic pH to favor acid tolerance [54,55]. Moreover, *Nhac* and *nhaK* genes, coding for sodium–proton (Na+/H+) antiporters and sodium, potassium, lithium, and rubidium/H(+) antiporters, maintaining pH and Na+ homeostasis, were present [56,57,58]. The alkaline shock response genes *sigW* and *rsiW* were also identified [59,60,61]. In addition, the *Mrp* (multiple resistance and pH) operon coding for seven hydrophobic gene products (*mrpA-mrpG*) was also observed [62]. Regarding bile salt resistance, mpr genes (A–G) [62], *ppaC*, which codes for inorganic pyrophosphatase (to maintain surface tension and keep membrane integrity) [63,64], *oppA* (oligopeptide-binding protein *OppA*) [65,66], and *ppaX* (pyrophosphatase) were observed [67]. Further, genes known to shield against osmotic stress environments (*opuD*, *opuE*, *opuAA*, *opuAB*, *opuAC*, *opuBA*, *opuBB*, *opuBC*, *opuBD*, *opuCA*, *opuCB*, *opuCC*, *opuCD*) were also seen in isolates [68,69,70]. The presence of these genes confirms the ability of the strain to adapt to an acidic environment.

The adherence ability of probiotic strains to the host epithelium is due to their cell surface proteins. The isolate genomes consisted of 11 genes that may encode adhesion-related proteins, including maltose phosphorylase (*mdxK*), sporulation and biofilm formation (*spo0A*) [71,72], lipoprotein signal peptidase I (*lspA*), elongation factor Tu (*tuf*) [73,74,75], sortase A (*srtA*) [76,77], and putative glycosyltransferase (*EpsH*) [78,79,80,81]. Another gene, *xylA*, linked with gut persistence was also found [80,82].

*B. subtilis* suffers from unavoidable oxidative stress during the exponential phase. Isolates harbor genes related to oxidative stress and, out of them, thioredoxin (*tpx*, *trxA*, *trxB*) [83,84,85], *BsaA* (glutathione peroxidase homolog) [86,87,88], and *ahpF* (NADH dehydrogenase) are antioxidant systems involved in ROS scavenging [89,90] (Table 2). The thioredoxin system can remove both ROS and RNS at a higher reaction rate by donating electrons to thiol-dependent peroxidases. The glutathione system detoxifies hydrogen peroxide and lipid peroxyl radicals by regulating the protein dithiol/disulfide balance [80]. In addition, genes for catalase (*KatA* and *KatE*) and glutaredoxin (*ytnI*) were also observed [91,92,93]. Other genes like *sodA* (superoxide dismutase [Mn]) [94,95,96], *sodF* (probable superoxide dismutase [Fe]) [97], *yojM* (superoxide dismutase-like protein) [98], and also the genes [manganese transport systems (*mntA-C*) and protein (*mntH*)] coding for the Mn^2+^ accumulation system were present [80]. Interestingly, methionine sulfoxide reductase genes (*msrA* and *msrB*) that can repair oxidized methionine residues by ROS in proteins were detected [99,100]. Moreover, the genes (*dlt* A–D) coding for immunomodulatory activities and some additional general stress genes were also identified [101]. These findings suggest that strains may withstand multiple stress conditions and are consistent with the adaptability characteristics of the gastrointestinal tract. Further, the adhesion-related protein contributes to the effective colonization of the intestinal environment and can eliminate unwanted gut microorganisms.

### 2.4. Genome Plasticity Analysis and Safety Assessment

#### Insertion Sequences

In the isolate BB10.1 genome, a total of eight insertion sequence (IS) elements belonging to two families (IS1182 and IS3) were predicted in the genome with a threshold E-value of 0.00001 (Supplementary Table S1). Among the predicted eight IS elements, six copies belonged to ISBpu1, one to ISBsu1, and one to ISBpe1. The isolate BP20.15 genome contained seven IS belonging to two families (one to IS3, and six to IS1182) (Supplementary Table S1). In the genome of isolate PY2.3, nine ISs were observed, all belonging to the IS3 family. Of the above-mentioned IS elements, only one of the insertion sequences (ISBsu1; IS3) from strain PY2.3 exhibited a score bit >1000 and an E-value of zero (Supplementary Table S1). However, none of the IS elements so far have been associated with safety risks. Moreover, in-depth analyses of genomes using Island Viewer 4 did not predict any virulence factors or pathogenicity-associated genes in any of the strains. The predicted genes for strains BB10.1 and BP20.15 were mapped to different genomic islands (Supplementary File S2). The majority of genes annotated from isolates genomes were hypothetical proteins, antioxidant genes, bacteriocins, insertion sequences, stress-related proteins, sporulation proteins, enzymes related to carbohydrate metabolism, transporters, etc., assisting the organism’s adaptability in the environmental niche [102]. No genomic islands were observed in the isolate PY2.3 genome.

The CRISPR-CasFinder tool identified three CRISPR arrays in the isolate BB10.1 genome; however, all the predicted arrays matched the consensus sequence with evidence level 1 (potentially invalid), and evidence level ≥3 is considered significant (Table 3). Five CRISPR arrays were identified in the genome of isolate BP20.15, but all the predicted arrays were disregarded as they were potentially invalid (evidence level 1). Moreover, no arrays with potential evidence were observed in the PY2.3 genome.

Full-length genome assemblies for each sample allowed for the prediction of the closest putative prophage (Table 4) using the PHASTER server. A total of six prophages were detected in the BB10.1 genome, which included one intact (region 3), one questionable (region 1), and four incomplete (regions 2, 4, 5, and 6) prophages (Table 4). Isolate BP20.15 yielded four incomplete prophage candidates. Whereas, in the PY2.3 genome, three prophage candidates were predicted: one intact (region 1), one questionable (region 2), and one incomplete (region 3). The intact phage (33.7 kb) of isolate BB10.1 (region 3; 662,637–696,369 bp) showed a maximum (46) protein matching and resembled PHAGE_Brevib_Osiris_NC_028969 (8). The other intact phage (31.7 kb) in isolate PY2.3 (region 1; 262,623–294,418 bp) showed a maximum (42) protein matching and resembled PHAGE_Brevib_Jimmer1_NC_029104 (8).

### 2.5. Safety-Associated Genes

The TABD v2 Wu-BLAST results were analyzed closely and most of the proteins in the genome of the isolates recognized by the database as toxins were predominantly the transporter proteins, other housekeeping proteins, and several hypothetical proteins. To search for the AMR genes, the Resfinder 4.1 database was used with default parameters (90% threshold and 60% minimum length). No AMR genes were detected in the genome of PY2.3; however, three AMR genes, *aadK* (streptomycin), *mph(K)* (spiramycin, telithromycin), and *tet(L)* (doxycycline, tetracycline), were detected in the genomes of isolates BB10.1 and BP20.15 (Table 5). 

The KEGG database search also yielded AMR-related genes in all three isolates’ genomes (Supplementary Table S2). In the genome of all the isolates (BB10.1, BP20.15, and PY2.3), the identified genes were related to vancomycin (*vanY*, *vanX*) (map01502), beta-lactam (*penP*) resistance (map01501), and cationic antimicrobial peptides (CAMPs) (map01503). Likewise, the CARD database search under default parameters (only perfect and strict hits) resulted in 18 hits (10 strict and 8 perfect hits) from the isolate BB10.1 genome. Isolate BP20.15 yielded 15 hits (14 strict hits and 1 perfect hit), and PY2.3 returned with 9 strict hits (Supplementary Table S3). The perfect hits in the BB10.1 genome had an identity of 100%, and the strict hits had an identity of 32–75% and included resistance genes to antibiotic target alteration (6), antibiotic target protection (1), antibiotic efflux (7), reduced permeability to antibiotics (1), and antibiotic inactivation (3). In isolate BB20.15, 14 strict hits (identity range 32–99%) and 1 perfect hit (100% identity) were found. The resistance genes included those for antibiotic target alteration (4), antibiotic target protection (1), antibiotic efflux (6), reduced permeability to antibiotics (1), and antibiotic inactivation (3). Whereas, in the genome of PY2.3, nine strict hits (identity 33–99%) were observed. The resistance genes included genes for antibiotic target alteration (4), antibiotic efflux (4), and antibiotic inactivation (1). Since the AMR genes of pathogenic bacteria are the major focus of both of the aforementioned databases, the AMR genes of non-pathogenic bacteria, such as *B. subtilis* and others, are often not incorporated in the databases.

VirulenceFinder detected no virulence genes under the BLASTn search. Sixty-nine (69) virulence genes were predicted by VFDB in the isolate BB10.1 genome, mainly associated with adherence, enzymes, immune evasion, iron acquisition, regulation, secretion system, and toxins (Supplementary Table S4). A total of 86 virulence genes were predicted by VFDB in the isolate BP20.15 genome, mainly associated with adherence, enzymes, immune evasion, iron acquisition, regulation, secretion system, toxin, acid resistance, anti-phagocytosis, copper uptake, iron uptake, peptidoglycan modification, stress adaptation, and surface protein anchoring (Supplementary Table S4). Seventy (70) virulence genes were predicted by VFDB in the isolate PY2.3 genome, mainly associated with adherence, enzymes, immune evasion, iron acquisition, regulation, secretion system, toxin, and cell surface components (Supplementary Table S4). The genes characterized as “virulence factors” in pathogens usually help them survive in the host environment under physiological stresses and can supposedly favor probiotic strain survival in the gut. OriTfinder did not detect any OriT in BB10.1 and BP20.15; however, in the genome of PY2.3, a 24 bp (AACCCCCCCACGCTAACAGGGGGG) DNA relaxase was observed having an 84% BlastP identity to the ICEBs1 mobile element of *B. subtilis*.

#### 2.5.1. Determination of Toxins, Biogenic Amines, and Undesirable Genes

The genes coding for undesirable properties were identified using BlastKoala and KAAS servers. Hemolysin (hlyIII; K11068, tlyc; K03699) was identified. Only L-lactate dehydrogenase (K00016) was present in all three strains and no gene for D-lactate dehydrogenase was found. 

Biogenic amine (BA) production is another essential trait related to probiotic safety issues. In all three genomes of isolates BB10.1, BP20.15, and PY2.3, genes related to spermidine synthase (*speE*, *SPE3*, *SRM*) [EC:2.5.1.16] (K00797; polyamine biosynthesis, arginine -> agmatine -> putrescine -> spermidine), arginine decarboxylase (*speA*) [EC:4.1.1.19] (K01585; polyamine biosynthesis, arginine -> agmatine -> putrescine -> spermidine), agmatinase (*speB*) [EC:3.5.3.11] (K01480; polyamine biosynthesis, agmatine -> putrescine), and arginase (E3.5.3.1, *rocF*, *arg*) [EC:3.5.3.1] (K01476; polyamine biosynthesis, arginine -> ornithine -> putrescine) were present.

**Figure 5:**
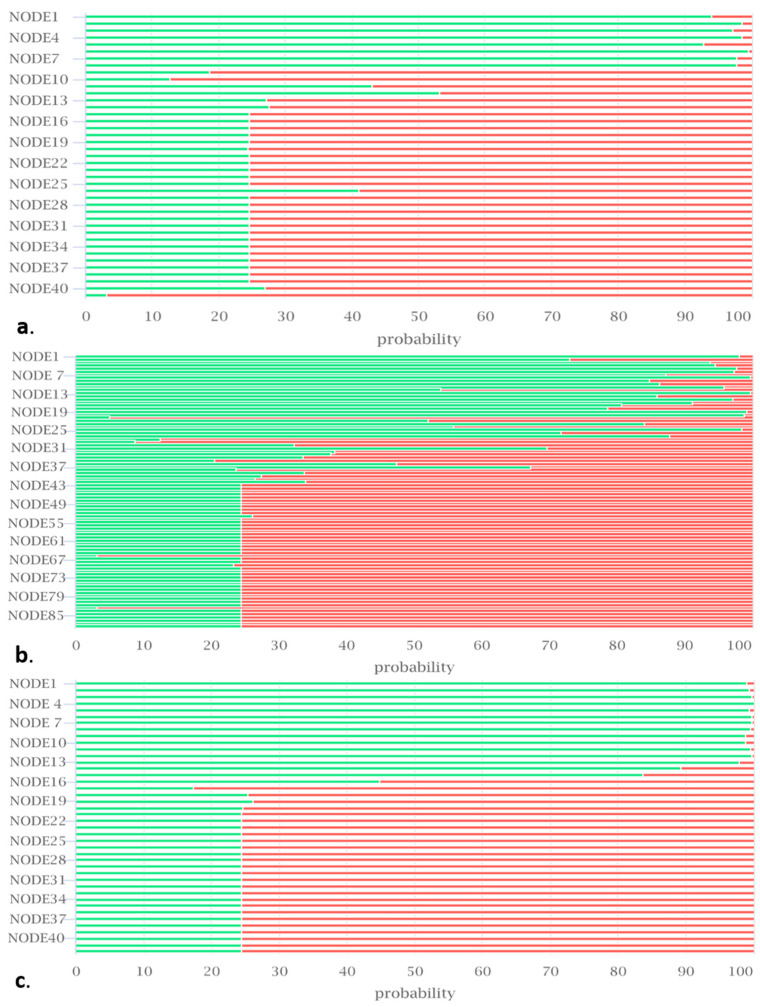
The predicted probability of probiotic genes in the genomes of isolates (**a**) BB10.1, (**b**) BP20.15, and (**c**) PY2.3. The green color indicates probiotic genes, and the red color indicates non-probiotic genes. The *x*-axis values (0–100) represent the probability of a gene being a probiotic gene. The *y*-axis represents the contig number in the genome.

Furthermore, no plasmids were detected in the genomes using the PlasmidFinder web tool [103], and the probability of being a human pathogen assessed using Pathogen Finder [104] was near zero (isolate BB10.1; 0.24; isolate BP20.15; 0.129; and isolate PY2.3; 0.118), indicating the safety of these strains. iProbiotics analysis revealed the portions of the genome of our isolates encoding for probiotics-properties-associated genes (Figure 5).

#### 2.5.2. Antimicrobial Peptides and Secondary Metabolites Analysis 

The Antismash tool detected 18 biosynthetic gene clusters (BGCs) in the BB10.1 genome, of which 13 encode antimicrobial peptides (Table 6). One cluster was found for bacillaene, subtilosin A, bacilysin, sporulation killing factor, and thailanstatin A. Two clusters for fengycin, plipastatin, and bacillibactin, and three were detected for surfactin. Additionally, one cluster encoding 1-carbapen-2-em-3-carboxylic acid and pulcherriminic acid was also identified. In the BP20.15 genome, 15 BCGs were detected (Supplementary Table S5a). One cluster was observed for fengycin, bacillaene, subtilosin A, bacilysin, sporulation killing factor, and bacillibactin. Two clusters were observed for surfactin and plipastatin. Similarly, one cluster encoding 1-carbapen-2-em-3-carboxylic acid and pulcherriminic acid was also identified. The PY2.3 genome revealed 13 BCGs (Supplementary Table S5b). One gene cluster was observed for macrolactin H, difficidin, fengycin, bacillaene, bacilysin, surfactin, bacillibactin, and plipastatin. Two clusters were observed for fengycin and terpene, and one for T3PKS. 

## 3. Discussion

In this study, the probiotic potential of *Bacillus* strains isolated from two essential natural bee products, bee bread (BB) and bee pollen (BP), was assessed [28]. BP and BB are increasingly gaining attention in the food industry as they can consist of rich macro- and micronutrients content with therapeutic properties, satisfying consumers’ trends for natural and functional foods.

The Generally Recognized As Safe (GRAS) status [105] of several *Bacillus* strains has increased their importance and role as probiotic starter cultures for developing functional foods with probiotic benefits for consumers [106,107,108,109]. However, before probiotic strains can exert their beneficial effects, they need to meet certain requirements. Based on the guidelines provided by the World Health Organization (WHO), some essential requirements for assessing a strain as an effective probiotic microorganism include their ability to tolerate gastrointestinal conditions, survive gastric acid and bile concentrations, adhere to intestinal epithelial cells, demonstrate antimicrobial actions, and lack antibiotic-resistant genes (FAO/WHO, 2006) [30]. Moreover, Chokesajjawatee et al., 2020 recommended some prerequisites for probiotic strains [110]. 

In pangenome analysis, the entire gene set of the selected *Bacillus* strains was compared, enabling the assessment of the genomic diversity of the entire repertoire of genes and the identification of core genomic elements [111]. The pan-genome is categorized into three categories: the core genome, the dispensable genome, and strain-specific unique genes [112]. To understand the relationships between pan-genome size, the core gene number, and the strain numbers, the fitted curves of the pan-genome profile were plotted for isolates. To determine the core genome, the number of conserved genes observed upon the sequential addition of new genomes was inferred by fitting a decaying function, implying that the average number of core genes converged to a relatively constant number. The core gene number in each genome varied slightly because of the involvement of duplicated genes and paralogs in the shared clusters. As illustrated in (Figure 2a,b), the blue curve increased with the addition of a new strain and was far from saturation, suggesting that the genetic repertoire of the species was nevertheless growing despite suitable adaptation to their diverse ecological niches. Thus, the pangenome of these strains was found to be open. This is in accordance with previous *Bacillus* pan-genome studies showing that environmental samples usually have open pan-genomes [113,114,115,116]. In other words, the isolated strains are expected to gain genes and evolve in the future. Thus, the availability of a large genetic repertoire might be an advantage for these strains to survive when facing environmental challenges. However, no novel genes were observed in any of our isolates that could confer any new functions, particularly in terms of safety and pathogenicity.

CAZyme profiling of the isolates revealed glycoside hydrolases (GHs) to be the most abundant CAZyme group. GH enzymes have significant potential to hydrolyze complex carbohydrates and are considered key enzymes in carbohydrate metabolism. They are commonly found in nature and can degrade abundant biomasses such as starch, cellulose, and hemicellulose [117]. An in-depth analysis for differentiating GH enzyme families in the isolates revealed the presence of several GH families, including GH1, GH101, GH105, GH11, GH126, GH13, GH13_11, GH13_23, GH13_29, GH13_3, GH13_30, GH13_31, GH16_21, GH171, GH18, GH23, GH24, GH26, GH28, GH3, GH30_3, GH30_8, GH32, GH36, GH38, GH4, GH42, GH43_11, GH43_4, GH43_5, GH46, GH5_11, GH51, GH53, GH65, GH68, GH73, and GH84. Some of these families are reported to be key enzymes in oligosaccharide degradation [118]. Oligosaccharides, as complex carbohydrates, are a major source of prebiotics, particularly galactans, and fructans, which have been linked to human gut health [119,120]. Galactans include galacto-oligosaccharides (GOSs), while fructans comprise fructo-oligosaccharides (FOSs) and inulin. Inulin, a well-known prebiotic, is a linear fructosyl polymer linked by β-(2,1) bonds (n = 3–65), attached to a terminal glucosyl residue by an α-(1,2) bond [121]. The hydrolysis of inulin is typically carried out by inulinase, an enzyme belonging to the GH32 family [122]. Interestingly, we detected genes related to GH32, which could be associated with inulinase production and its strong ability to consume inulin [123]. Moreover, it also suggests that the presence of these enzymes can positively affect the availability of bee pollen and bee bread compounds for young bees and humans.

Glycosyltransferases (GTs) were the second most abundant group, and the cellulose synthase GT2, an important enzyme for cellulose biosynthesis, was identified in the genomes of all three isolates. It stores cellulose on the cell wall surface as an extracellular matrix for cell adhesion and biofilm formation, providing protection from the surrounding environment [124]. Glycosyltransferases catalyze the transfer of sugars from activated donor molecules to specific acceptors, playing an essential role in the formation of surface structures recognized by host immune systems [125]. Analysis by the dbCAN server also revealed the presence of carbohydrate binding modules (CBMs) in all the isolates. CBM enzymes are mostly associated with GHs, binding to carbohydrate ligands and enhancing the catalytic efficiency of carbohydrate-active enzymes [126]. In BB10.1, a total of four auxiliary activity (AA) enzymes were observed, consisting of one from AA4, two from AA6, and one from AA7. Similarly, in BP20.15, four AA enzymes were identified, comprising one from family AA4, two from AA6, and one from AA7. Additionally, in PY2.3, a single auxiliary activity (AA) enzyme, AA10, was observed. Therefore, the high number of GH and GT genes, along with other CAZyme genes in these strains, suggests their probiotic potential, particularly for immune stimulation, pathogen defense, and the production of essential fermentation end-products in fermented foods.

KEGG analysis revealed that most of the genes in BB10.1, BP20.15, and PY2.3 are involved in genetic information processing, carbohydrate metabolism, protein signaling and cellular processes, environmental information processing, amino acid metabolism, nucleotide metabolism, and, notably, the metabolism of cofactors and vitamins. BlastKOALA revealed genes related to riboflavin metabolism, vitamin B6 metabolism, biotin metabolism, and folate biosynthesis. The gut microbiota plays a crucial role in aiding the host by contributing to nutrient digestion and energy recovery [100]. The capacity to produce folate has been investigated in various probiotic strains due to its potentially relevant applications [127,128]. Previous studies have shown that the *B. subtilis* genome contains all the pathways and components necessary for folate biosynthesis and has been engineered for folate production [128,129,130].

Probiotic strains encounter various harsh environmental conditions during transport in the gastrointestinal tract, including the acidic conditions of the stomach, the bile juice environment in the small intestine, oxidative stress, and osmotic stress [131]. The F0F1 ATP synthase pump helps to maintain H+ homeostasis when bacteria face an acidic environment. It hydrolyzes ATP to pump protons (H+) from the cytoplasm [132]. We found that this synthase complex is present in the genomes of our isolates. Bacteria must withstand the toxicity of bile salts, which induce intracellular acidification and act as detergents that disrupt biological membranes [133]. Proteins involved in bile tolerance mechanisms, such as the Na(+)/H(+) antiporter, manganese-dependent inorganic pyrophosphatase, pyrophosphatase PpaX, and oligopeptide-binding protein OppA, were also identified. Molecular chaperones that impart resistance against environmental stress, such as the chaperonins GroES and GroEL [134,135], six Clp proteases, and HtpX protease heat shock proteins, were present. Three copies of cold shock proteins (CSPs) were also found. These proteins play important roles in basic cellular functions, including growth, DNA and RNA stability, and the prevention of inclusion body formation [136,137,138].

To handle hyperosmotic stress and heat resistance, the isolates possess one copy of the chaperone protein DnaJ, the chaperone protein DnaK, and the nucleotide exchange factor GrpE. Additionally, two methionine sulfoxide reductases [139], along with others, were present in the genomes of the isolates, providing resistance to oxidative stress. Annotation analysis also revealed the presence of proteins involved in adhesion. These adhesion proteins may facilitate the binding of probiotic bacteria and enable direct interactions with the intestinal mucosa layer. All the isolates also harbored general stress adaptation proteins. Universal stress proteins are important for survival during cellular growth arrest and help to reprogram the cell toward defense and escape during cellular stress [140,141]. This suggests that our isolates have proteins that can handle stress and harsh conditions in the human gut and improve adhesion to the intestinal mucosa.

Concerning the safety assessment, KEGG analysis revealed the presence of hemolysin (*hlyIII*) in the genomes of all three strains. However, our wet lab studies found the activity to be γ-hemolysis [142], which is generally considered safe [44,143]. Moreover, the KEGG analysis indicated antibiotic resistance to vancomycin (*vanY*, *vanX*), beta-lactam (*penP*), and cationic antimicrobial peptides. Resfinder 4.1 predicted streptomycin, spiramycin/telithromycin, and doxycycline/tetracycline resistance in the isolates BB10.1 and BP20.15. Nevertheless, in the antibiotic susceptibility tests conducted in the wet lab with chloramphenicol, azithromycin, linezolid, rifampicin, penicillin, trimethoprim, clindamycin, ciprofloxacin, gentamycin, kanamycin, and streptomycin (results not shown), resistance was observed only for penicillin. While tetracycline- and vancomycin-resistant genes were predicted computationally, we did not perform any susceptibility studies to substantiate or confirm any intrinsic resistance. Different classes of beta-lactamase presence in one or the other *Bacillus* probiotic suggest the presence of penicillin resistance in the *Bacillus* probiotic [100,144]. Moreover, previous studies have shown that an organism may exhibit intrinsic resistance to a few antibiotics that cannot be attributed to its genotype [144].

Conjugative elements and phage-mediated insertions play significant roles in bacterial evolution [145] by contributing to genetic variability among closely related bacterial strains [146]. This variability often leads to phenotypical differences, such as in bacterial pathogenesis [146,147]. Bacteriophage-mediated horizontal gene transfer enhances bacterial adaptive responses to environmental changes, including the rapid spread of antibiotic resistance [148]. Furthermore, phages facilitate inversions, deletions, and chromosomal rearrangements, which help to transfer genes that can directly impact the phenotype between related or phylogenetically distant strains through horizontal gene transfer (HGT), and all of these evolutionary events have implications for selection and fitness [146,147]. Brevibacillus phage Osiris, a temperate phage, was reported in the genome of isolate BB10.1 [149]. The BB10.1 genome had a GC content of 43.67%, and the Brevibacillus phage Osiris had a GC content of 44.85%. Similarly, Brevibacillus phage Jimmer 1, another temperate phage reported in the genome of isolate PY2.3, specifically targets *Paenibacillus larvae*, a Firmicute bacterium, as its host [150]. Isolate PY2.3 had a GC content of 46.52%, while the Brevibacillus phage Jimmer1 region had a GC content of 47.00%. The distribution of phage elements is not consistently associated with resistance properties. The Jimmer1 phage includes many genes found in all strains, whether resistant or not [151]. Incomplete prophage regions are considered as defective prophages, lacking complete structural prophage genes compared to active, functional phages. Moreover, defective prophages often carry genes beneficial to the host, such as recombination, virulence, stress resistance, or toxin genes, that can inhibit the growth of competing bacteria in the environment [152]. 

Insertion sequences (ISs) are short DNA sequences that function as simple transposable elements. They are often smaller than other transposable elements and encode only proteins involved in transposition [153]. In the genomes of isolates BB10.1 and BP20.15, copies of the insertion sequences ISBsu1 and ISBpu1 were found, while BB10.1 also had a copy of ISBspe1. PY2.3, along with copies of ISBsu1, also had one copy of IS665 and ISLmo1. These elements, along with others, have been reported in several other *Bacillus* species [154,155,156,157]. However, none of these elements have been associated with pathogenicity or virulence in *Bacillus* spp., but they have been linked to stress tolerance [158]. ICEs (integrative and conjugative elements) are mobile genetic elements that significantly contribute to genome evolution [159]. In the genome of isolate PY2.3, a mobile genetic element called ICEBs1 was found. ICEBs1 is an integral part of the global DNA damage SOS response and has also been reported in other *Bacillus* genomes [160]. It has been associated with gene activation and inactivation, recombination and rearrangement, evolution of new functions, antibiotic resistance and virulence, horizontal gene transfer, adaptation to environmental changes, stress response, resistance to radiation, and desiccation [151].

Biogenic amines (BAs) are naturally occurring low-molecular-weight organic nitrogen bases found in living organisms. These compounds serve as metabolic intermediates and products, synthesized and degraded during the metabolism of animals, plants, and microorganisms. BAs are primarily formed through the decarboxylation of amino acids or the amination and transamination of aldehydes and ketones [161,162]. They possess chemical structures that can be classified as aliphatic (such as putrescine, cadaverine, spermine, and spermidine), aromatic (tyramine and phenylethylamine), or heterocyclic (histamine and tryptamine) [163]. BAs play critical roles in various human physiological functions, including cerebral activity, gastric acid secretion, and immune responses [164]. The accumulation of BAs in food can occur at high concentrations due to the activities of microorganisms possessing decarboxylation enzymes. Excessive oral intake of BAs can result in symptoms such as nausea, headaches, rashes, and changes in blood pressure [164]. Consequently, it is essential to prevent BA accumulation in food to avoid adverse health effects [165]. In our isolates, genes associated with the synthesis of spermidine and putrescine were detected. Spermidine plays a critical role in the robust formation of biofilms in *B. subtilis* [166]. In *B. subtilis*, the triamine spermidine is formed from the diamine putrescine through the transfer of an aminopropyl group to putrescine by spermidine synthase, which is encoded by the *speE* gene [166]. However, genes related to other biogenic amines (cadaverine, ornithine, histamine, tyramine, and tryptamine) were not detected in any of the isolate genomes, as described by Chokesajjawatee et al., 2020 [110].

## 4. Materials and Methods

### 4.1. Genomic Sequences

The genomic material in this in silico investigation comprised three *Bacillus* strains: BB10.1 and BP20.15 isolated from bee bread, and PY2.3 obtained from bee pollen. These genomic sequences were publicly accessible via the Sequence Read Archive (SRA) and GenBank, associated with BioProject IDs PRJNA949953, PRJNA949979, and PRJNA949984, respectively. The SRA accession numbers for BB10.1, BP20.15, and PY2.3 were cataloged as SRR24003043, SRR24003725, and SRR24005238, respectively [28].

### 4.2. Genome Synteny and Completeness 

To assess the completeness of the genome assembly and the quality of the protein annotation, Benchmarking Universal Single-Copy Orthologs (BUSCO) tool 5.4.6 [167] with bacilli_odb10 as reference lineage dataset was used, and D-Genies web server4 [168] was used to evaluate bacterial genome synteny. Dot plots generated were used for visual analysis. 

### 4.3. Pan-Genome Analysis 

To obtain an insight into the genomes of other isolates, pan-genome analysis was performed with 180 *B. subtilis* genomes and 204 *Bacillus velezensis* genomes (accession number Supplementary File S1) using IPGA v1.09 (integrated prokaryotes genome and pan-genome analysis service) [169]. The following parameters were used: genome filter; completeness 90; contamination 5; and, for pan-genome analysis procedures, modules, PANOCT, OrthoMCL, Roary, panX, OrthoFinder, Panaroo, and PPanGGoLiN were selected. Further downstream analyses were performed in the R studio using R modules ggplot2 and heatmap.2. Whole-genome-variation-based phylogenetic inference tree was generated and the binary matrix file, gene presence, and absence across all strains were used to estimate the sizes of the pan-genome and core genome.

### 4.4. Probiogenomics Analysis

#### 4.4.1. Functional Annotation and Carbohydrate-Active Enzyme (CAZyme) Profiling 

The Kyoto Encyclopedia of Genes and Genomes (KEGG) Database and Links Annotation (BlastKOALA) (https://www.kegg.jp/blastkoala/ (accessed on 5 August 2023)) and EGGNOG2-mapper V2 [170] were used for the functional annotation of the proteins. Carbohydrate-active enzymes (CAZymes) were annotated by the dbCAN database (https://bcb.unl.edu/dbCAN2/index.php (accessed on 5 August 2023)) [171], a Meta server for automated carbohydrate-active enzyme annotation. CAZymes predicted by any two of the tools out of three were considered.

#### 4.4.2. Genome Plasticity Analysis and Safety Assessment 

To analyze the genome plasticity, the web server PHASTER was used for the rapid identification and annotation of prophage sequences within bacterial genomes [172]. The insertion elements in the genome were detected with the ISfinder database [173] using BLASTn v2.2.31 with an E-value threshold of 1 × 10^−5^. The Island Viewer 4 server was used for determining the genomic islands and the presence of genes related to pathogenicity [174]. The CRISPRCasFinder tool was used to determine the sequences coding for clustered regularly interspaced short palindromic repeats (CRISPRs) and CRISPR-associated genes (Cas) using default parameters [175]. To detect the presence of plasmids in the genome, the tool Plasmid Finder was used [103]. To search for the toxin–antitoxin proteins in the genomes of our selected strains, a WU-BLAST 2.0 search was carried out against the TADB v2.0 finder database [176] with the following parameters: (1) E-value for BLAST = 0.01, (2) E-value for HMMer = 1, (3) maximum length of potential toxin/antitoxin = 300, (4) maximum distance (or overlap) between potential toxin and antitoxin = 20–150 nucleotides. The comparison matrix was BLOSUM62, the cutoff score (S value) was default, the word length (W value) was default (default = 11 for BLASTN, 3 for all others), and the expected threshold (E threshold) was default.

Subsequently, to assess the safety of isolates, the search for antimicrobial resistance (AMR) genes in the genomes of isolates BB10.1, BP20.15, and PY2.3 was carried out in three publicly available databases, i.e., ResFinder tool v.4.1. of the Center for Genomic Epidemiology [177], the Resistance Gene Identifier (RGI) tool in the Comprehensive Antibiotic Resistance Database (CARD) [178], and the BlastKOALA and KAAS tool in the KEGG database [179]. The Virulence Finder v.2.0.3 tool (https://cge.food.dtu.dk/services/VirulenceFinder/ (accessed on 7 August 2023)) and Virulence Factor of Bacterial Pathogen Database (VFDB) [180] were used to determine the putative virulence factors in the compare draft genome of isolates with known *Bacillus* pathogens. A web-based tool oriTfinder (available at https://bioinfo-mml.sjtu.edu.cn/oriTfinder/ (accessed on 7 August 2023)) [181] was used to identify the origin of transfer (oriT), the essential element for self-transmitted conjugative plasmids. The results from the BlastKOALA and KAAS tool in the Kyoto Encyclopedia of Genes and Genomes (KEGG) database were critically observed, and the genes involved in toxins, biogenic amine (BA) production, and other undesirable properties as stated by Chokesajjawatee et al., 2020 [110] were determined by literature mining and annotated genome analyses. PathogenFinder [104] was used for assessing the probability of the isolates being pathogenic to humans. iProbiotics (http://bioinfor.imu.edu.cn/iprobiotics/public/Home (accessed on 15 August 2023)) [182], a web-based machine learning tool for the detection of probiotics genes, was used to identify probiotics genes in our isolates genomes.

## 5. Conclusions

We have identified several promising probiotic characteristics in the genomes of our isolates. It is essential to conduct in vitro and in vivo studies to confirm and further investigate specific traits, such as the potential production of biogenic amines. Additionally, more extensive investigations are needed to understand the mechanisms underlying tetracycline and vancomycin resistance, including the role of insertion elements, which warrants a broader range of molecular biology studies. Furthermore, while the iProbiotics tool successfully identified potential probiotic regions, it lacks additional information regarding non-probiotic regions. Specifically, details about the presence of genes contributing to virulence and the similarity of these regions to other pathogenic strains are not provided. This information could be of considerable significance in evaluating the characteristics of non-probiotic regions.

## Figures and Tables

**Table 1:** BUSCO assembly analysis for strains BB10.1, BP20.15, and PY2.3 assemblies.

BUSCO Groups Searched against Bacilli_odb10 Lineage	BB10.1	BP20.15	PY2.3
Complete BUSCOs (C)	449 (99.6%)	448 (99.5%)	302 (100.0%)
Complete and single-copy BUSCOs (S)	448 (99.6%)	447 (99.3%)	301 (99.7%)
Complete and duplicated BUSCOs (D)	1 (0.2%)	1 (0.2%)	1 (0.3%)
Fragmented BUSCOs (F)	1 (0.2%)	2 (0.4%)	0 (0.0%)
Missing BUSCOs (M)	0 (0%)	0 (0.1%)	0 (0.0%)
Total BUSCO groups searched	450	450	302

**Table 2:** Stress response genes mined from BB10.1, BP20.15, and PY2.3 annotated genomes.

Gene	Product Description	Uniprot ID
Heat stress		
*htpX*	Protease HtpX homolog; heat shock protein	O31657
*hrcA*	Heat-inducible transcription repressor	P25499
*hslO*	33 kDa chaperonin	P37565
*dnaK*	Chaperone protein DnaK	P17820
*dnaJ*	Chaperone protein DnaJ	P17631
*ctsR*	Transcriptional regulator of stress and heat shock response	P37568
*grpE*	Olecular chaperone GrpE	P15874
*groL*	60 kDa chaperonin GroEL	P28598
*groS*	10 kDa chaperonin GroES	A7Z206
*lon1*	ATP-dependent Lon protease	P37945
*lon2*	ATP-dependent Lon protease	P42425
*clpC*	ATP-dependent Clp protease ATP-binding subunit ClpC	P37571
*clpE*	ATP-dependent Clp protease ATP-binding subunit ClpE	O31673
*clpP*	ATP-dependent Clp protease proteolytic subunit clpP	P80244
*clpQ*	ATP-dependent protease subunit ClpQ	P39070
*clpX*	ATP-dependent Clp protease ATP-binding subunit ClpX	P50866
*clpY*	ATP-dependent protease ATPase subunit ClpY	P39778
Cold		
*cspB*	Cold shock protein CspB	P32081
*cspC*	Cold shock protein CspC	P39158
*cspD*	Cold shock protein CspD	P51777
Acid stress		
*atpA*	ATP synthase subunit alpha	P37808
*atpB*	ATP synthase subunit beta	P37809
*atpC*	ATP synthase epsilon chain	P37812
*atpD*	ATP synthase subunit beta	P37809
*atpE*	ATP synthase subunit c	A7Z9Q5
*atpF*	ATP synthase subunit b	P37814
*atpG*	ATP synthase gamma chain	P37810
*atpH*	ATP synthase subunit delta	P37811
*atpI*	ATP synthase protein I	P37816
*nhaC*	Na(+)/H(+) antiporter NhaC	O07553
*nhaK*	Sodium, potassium, lithium, and rubidium/H(+) antiporter	O32212
*nhaX*	Stress response protein NhaX	O07552
*sigW*	RNA polymerase sigma factor SigW	Q45585
*rsiW*	Anti-sigma-W factor RsiW	Q45588
Bile tolerance		
*mrpA*	Na(+)/H(+) antiporter subunit A	Q9K2S2
*mrpB*	Na(+)/H(+) antiporter subunit B	O05259
*mrpC*	Na(+)/H(+) antiporter subunit C	O05260
*mrpD*	Na(+)/H(+) antiporter subunit D	O05229
*mrpE*	Na(+)/H(+) antiporter subunit E	Q7WY60
*mrpF*	Na(+)/H(+) antiporter subunit F	O05228
*mrpG*	Na(+)/H(+) antiporter subunit G	O05227
*ppaC*	Manganese-dependent inorganic pyrophosphatase	P37487
*ppaX*	Pyrophosphatase PpaX	Q9JMQ2
*oppA*	Oligopeptide-binding protein OppA	P24141
Osmoprotectant		
*opuD*	Glycine betaine transporter OpuD	P54417
*opuBD*	Choline transport system permease protein OpuBD	P39775
*opuBC*	Choline-binding protein	Q45462
*opuBB*	Choline transport system permease protein OpuBB	Q45461
*opuBA*	Choline transport ATP-binding protein OpuBA	Q45460
*opuCD*	Glycine betaine/carnitine/choline transport system permease protein OpuCD	O34742
*opuCC*	Glycine betaine/carnitine/choline-binding protein OpuCC	O32243
*opuCB*	Glycine betaine/carnitine/choline transport system permease protein OpuCB	O34878
*opuCA*	Glycine betaine/carnitine/choline transport ATP-binding protein OpuCA	O34992
*opuE*	Osmoregulated proline transporter OpuE	O06493
*opuAA*	Glycine betaine transport ATP-binding protein OpuAA	P46920
*opuAB*	Glycine betaine transport system permease protein OpuAB	P46921
*opuAC*	Glycine betaine-binding protein OpuAC	P46922
Adhesion		
*lspA*	Lipoprotein signal peptidase	Q45479
*spo0A*	Stage 0 sporulation protein A	P06534
*Tuf*	Elongation factor Tu	P33166
*tpiA*	Triosephosphate isomerase	P27876
*gapA*	Glyceraldehyde-3-phosphate dehydrogenase 1	P09124
*ganA*	Beta-galactosidase GanA	O07012
*srtD*	Sortase D	P54603
*mdxK*	Maltose phosphorylase	O06993
*Eno*	Enolase	P37869
*Pgi*	Glucose-6-phosphate isomerase	P80860
*EpsH*	Putative glycosyltransferase	P71057
Antioxidant		
*katA*	Vegetative catalase	P26901
*katE*	Catalase-2	P42234
*fnr*	Anaerobic regulatory protein	P46908
*ytnI*	Putative glutaredoxin YtnI	O34639
*ggt*	Glutathione hydrolase proenzyme	P54422
*bsaA*	Glutathione peroxidase homolog BsaA	P52035
*mntH*	Divalent metal cation transporter MntH	P96593
*mntD*	Manganese transport system membrane protein	O34500
*mntC*	Manganese transport system membrane protein	O35024
*mntB*	Manganese transport system ATP-binding protein	O34338
*mntA*	Manganese-binding lipoprotein	O34385
*ahpF*	NADH dehydrogenase	P42974
*tpx*	Thiol peroxidase	P80864
*trxA*	Thioredoxin	P14949
*trxB*	Thioredoxin reductase	P80880
*msrA*	Peptide methionine sulfoxide reductase	P54154
*msrB*	Peptide methionine sulfoxide reductase	P54155
*sodF*	Probable superoxide dismutase [Fe]	O35023
*sodA*	Superoxide dismutase [Mn]	P54375
*yojM*	Superoxide dismutase-like protein	O31851
*ytnI*	Putative glutaredoxin YtnI	O34639
Immunomodulation		
*dltA*	Alanine–D-alanyl carrier protein ligase	P39581
*dltB*	Teichoic acid D-alanyltransferase	P39580
*dltC*	Alanyl carrier protein	P39579
*dltD*	Protein DltD	P39578
Additional stress response genes		
*ykoL*	Stress response protein YKoL	O34763
*yhaX*	Stress response protein YhaX	O07539
*nhaX*	Stress response protein NhaX	O07552
*yhbH*	Stress response UPF0229 protein YhbH	P45742
*ctc*	General stress protein CTC	P14194
*yocK*	General stress protein 16O	P80872
*yocM*	Salt-stress-responsive protein YocM	O34321
*ysnF*	Stress response protein YsnF	P94560
*dps*	General stress protein 20U	P80879
*yugI*	General stress protein 13	P80870
*mrgA*	Metalloregulation DNA-binding stress protein	P37960
*yvgO*	Stress response protein YvgO	O32211
*ywrO*	General stress protein 14	P80871
*csbD*	Stress response protein CsbD	P70964
*yfkM*	General stress protein 18	P80876
*yflT*	General stress protein 17M	P80241
*gspA*	General stress protein A	P25148
*yxiE*	Universal stress protein YxiE	P42297
*ydaD*	General stress protein 39	P80873

**Table 3:** Crispr array and Cas type detection within BB10.1, BP20.15, and PY2.3 genomes using CRISPRFinder tool.

CRISPR Id/Cas Type	Start	End	Spacer/ Gene	Repeat Consensus/ Cas Genes	Repeat Length	No. of CRISPRs with Same Repeat (Crisprdb)	Direction	Evidence Level
Isolate BB10.1								
NODE2_*B. subtilis*_chromosome	1,042,827	1,042,934	1	TGATGGGAATCGAACCCACGACAT	24	0	ND	1
NODE3_*B. subtilis*_chromosome	536,583	536,695	1	GAAGATTTTAGTGATCGTTTAGATGATTTTGA	32	0	ND	1
NODE4_*B. subtilis*_chromosome	8684	8789	1	CAGCTGATTGCTGGTTTTGTTTTCT	25	0	ND	1
Isolate BP20.15								
NODE6_*B. subtilis*_chromosome	72,057	72,169	1	GAAGATTTTAGTGATCGTTTAGATGATTTTGA	32	0	ND	1
NODE8_*B. subtilis*_chromosome	64,916	65,023	1	TGATGGGAATCGAACCCACGACAT	24	0	ND	1
NODE25_*B. subtilis*_chromosome	8546	8651	1	CAGCTGATTGCTGGTTTTGTTTTCT	25	0	ND	1
NODE41_CAS type	3600	4991	2	cas3_TypeI, cas3_TypeI	--	--	--	--
NODE75_*B. subtilis*_chromosome	55	162	1	GTCGCAATTGCATCCACTTTACTCATG	27	0	ND	1
Isolate PY2.3								
NODE13_*B. velezensis*_CAS cluster	45,589	46,974	2	cas3_TypeI, cas3_TypeI	--	--	--	--

**Table 4:** Prophage regions of isolates BB10.1, BP20.15, and PY2.3 identified by the PHASTER tool.

Region	Region Length	Completeness	Score	Total Proteins	Region Position	Most Common Phage	GC%
Isolate 10.1							
1	32.4 Kb	questionable	80	44	84,936–117,366	PHAGE_Bacill_phi105_NC_048631(16)	40.13%
2	31.7 Kb	incomplete	30	15	107,047–138,752	PHAGE_Bacill_BM5_NC_029069(4)	39.18%
3	33.7 Kb	intact	110	46	662,637–696,369	PHAGE_Brevib_Osiris_NC_028969(8)	44.85%
4	9.5 Kb	incomplete	10	18	60,849–70,358	PHAGE_Bacill_SPbeta_NC_001884(7)	33.61%
5	40.6 Kb	incomplete	40	55	531,669–572,327	PHAGE_Bacill_vB_BtS_BMBtp14_NC_048640(7)	39.50%
6	11.7 Kb	incomplete	10	15	270,249–282,042	PHAGE_Thermu_OH2_NC_021784(2)	45.09%
Isolate 20.15							
1	25.7 Kb	incomplete	40	35	230,224–255,996	PHAGE_Bacill_SPP1_NC_004166(13)	41.94%
2	11.7 Kb	incomplete	10	15	5540–17,317	PHAGE_Thermu_OH2_NC_021784(2)	45.18%
3	20.3 Kb	incomplete	20	28	32,616–52,954	PHAGE_Brevib_Osiris_NC_028969(5)	45.48%
4	19.4 Kb	incomplete	30	32	1–19,441	PHAGE_Anoxyb_A403_NC_048701(4)	42.37%
Isolate PY2.3							
1	31.7 Kb	intact	100	42	262,623–294,418	PHAGE_Brevib_Jimmer1_NC_029104(8)	47.00%
2	47.4 Kb	questionable	70	57	334,721–382,135	PHAGE_Bacill_SPP1_NC_004166(15)	41.90%
3	23 Kb	incomplete	20	15	58,603–81,625	PHAGE_Clostr_phi3626_NC_003524(2)	37.85%

**Table 5:** AMR (antimicrobial resistance) genes identified and their location in the isolates’ genomes.

Resistance Gene	Identity%	Alignment Length/Gene Length	Position in Reference	Contig or Depth	Position in Contig	Phenotype	PMID	Accession No.
Isolate BB10.1								
*aadK*	100.0	855/855	1..855	NODE2	606,252–607,106	streptomycin	2550327	M26879
*mph(K)*	100.0	921/921	1..921	NODE7	99,651–100,571	spiramycin, telithromycin	29317655	NC_000964
*tet(L)*	100.0	1377/1377	1..1377	NODE8	90,607–91,983	doxycycline, tetracycline	2844262	X08034
Isolate BP20.15								
*aadK*	100.0	855/855	1..855	NODE2	606,252–607,106	streptomycin	2550327	M26879
*tet(L)*	100.0	1377/1377	1..1377	NODE8	90,607–91,983	doxycycline, tetracycline	2844262	X08034
*mph(K)*	100.0	921/921	1..921	NODE7	99,651–100,571	spiramycin, telithromycin	29317655	NC_000964

**Table 6:** List of identified secondary metabolite clusters of isolate BB10.1 using strictness ‘strict’.

Region	Type	From	To	Most Similar Known Cluster	Similarity
Region 1.1	NRPS, betalactone	1	27,989	Fengycin; NRP	86%
Region 1.2	NRPS, transAT-PKS, T3PKS	141,809	247,055	Bacillaene; Polyketide + NRP	100%
Region 1.3	terpene	840,077	860,880	---	---
Region 2.1	NRPS	1	22,938	Plipastatin; NRP	38%
Region 2.2	terpene	97,150	119,048	---	---
Region 2.3	T3PKS	167,536	208,633	1-carbapen-2-em-3-carboxylic acid; Other	16%
Region 3.1	NRP-metallophore, NRPS	82,076	133,853	Bacillibactin; NRP	100%
Region 3.1	NRP-metallophore, NRPS	82,076	133,853	Bacillibactin; NRP	100%
Region 3.2	CDPS	415,378	436,124	Pulcherriminic acid; Other	100%
Region 3.3	Sactipeptide	647,652	669,263	Subtilosin A; RiPP:Thiopeptide	100%
Region 5.1	other	1	35,524	Bacilysin; Other	100%
Region 6.1	NRPS	1	26,533	Surfactin; NRP:Lipopeptide	43%
Region 7.1	sactipeptide, ranthipeptide	27,754	50,707	Sporulation killing factorRiPP:Head-to-tailcyclized peptide	100%
Region 7.2	NRPS	180,780	208,793	Surfactin; NRP:Lipopeptide	43%
Region 8.1	epipeptide	18,667	40,365	Thailanstatin A; NRP + Polyketide	10%
Region 12.1	NRPS	1	14,431	Fengycin; NRP	20%
Region 13.1	NRPS	1	11,532	Plipastatin; NRP	23%
Region 14.1	NRPS	1	10,548	Surfactin; NRP:Lipopeptide	8%

## Data Availability

The data presented in this study are available on request from the corresponding author.

## Supplementary Materials

The following supporting information can be downloaded at: https://www.mdpi.com/article/10.3390/ijms25010666/s1.
