# Supplementary material for: In Silico Safety Assessment of Bacillus Isolated from Polish Bee Pollen and Bee Bread as Novel Probiotic Candidates
Source: Int J Mol Sci. 2024 Jan 4;25(1):666. doi: 10.3390/ijms25010666 (PMC10780176; doi:10.3390/ijms25010666)
Supplement: Supplementary file 1 [file ijms-25-00666-s001.zip › Supplemetary figures_In Silico Safety Assessment of Bacillus isolated from Polish Bee Pollen and Bee Bread as Novel Probiotic Candidates.pdf]

In Silico Safety Assessment of *Bacillus* isolated from Polish Bee Pollen and Bee Bread as Novel Probiotic Candidates

Ahmer Bin Hafeez[1]; Karolina Pełka[1]; Randy Worobo[2]; Piotr Szweda[1*]

1 - Department of Pharmaceutical Technology and Biochemistry, Faculty of Chemistry, Gdańsk University of Technology, ul. G. Narutowicza 11/12, 80-233 Gdańsk, Poland;

2 - Department of Food Science, Cornell University, Ithaca, NY 14853, USA

Ahmer Bin Hafeez – e-mail: ahmer.bin.hafeez@pg.edu.pl; ORCID: 0000-0002-8712-5898

Karolina Pełka – e-mail: karolina.pelka@pg.edu.pl; ORCID: 0000-0003-2523-8165

Randy Worobo – e-mail: rww8@cornell.edu; ORCID: 0000-0002-5211-3125

Piotr Szweda – email: piotr.szweda@pg.edu.pl; ORCID: 0000-0001-8291-5148[*]

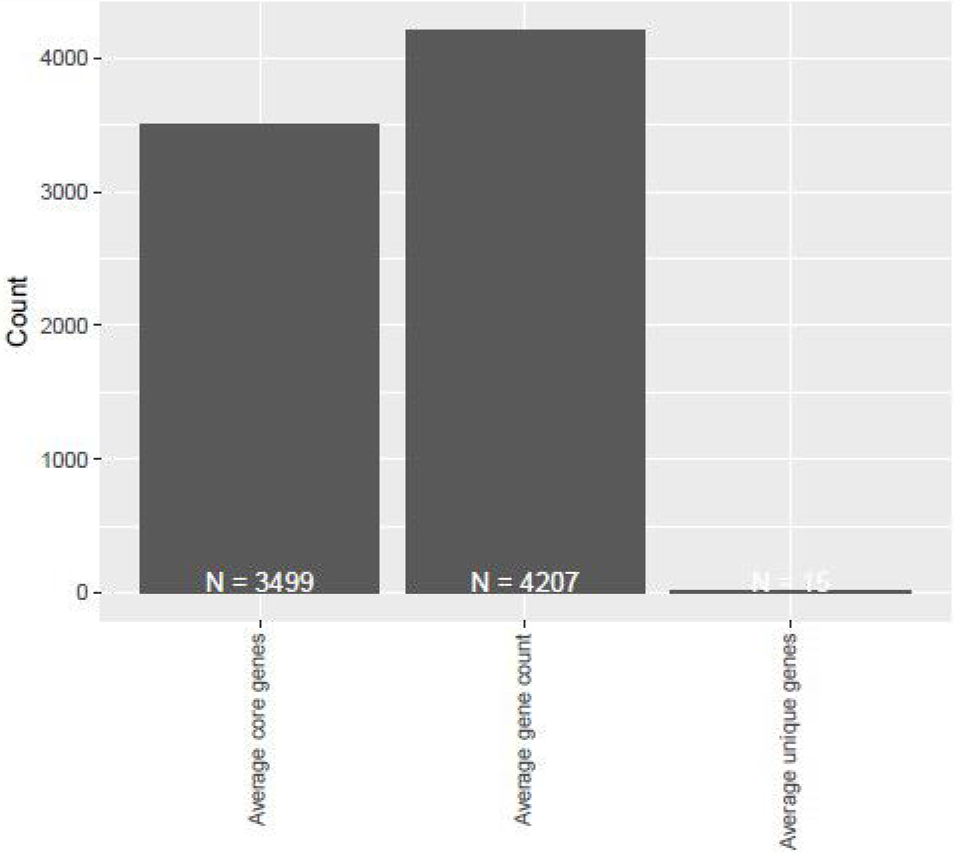

Total number of genomes:
180

Number of analyzed genes:
752925

Total orthologous groups
10810

Total unique genes
2664

Figure S1. Number of Average core genes, average gene count, and average unique genes present in the *B. subtilis* pangenome

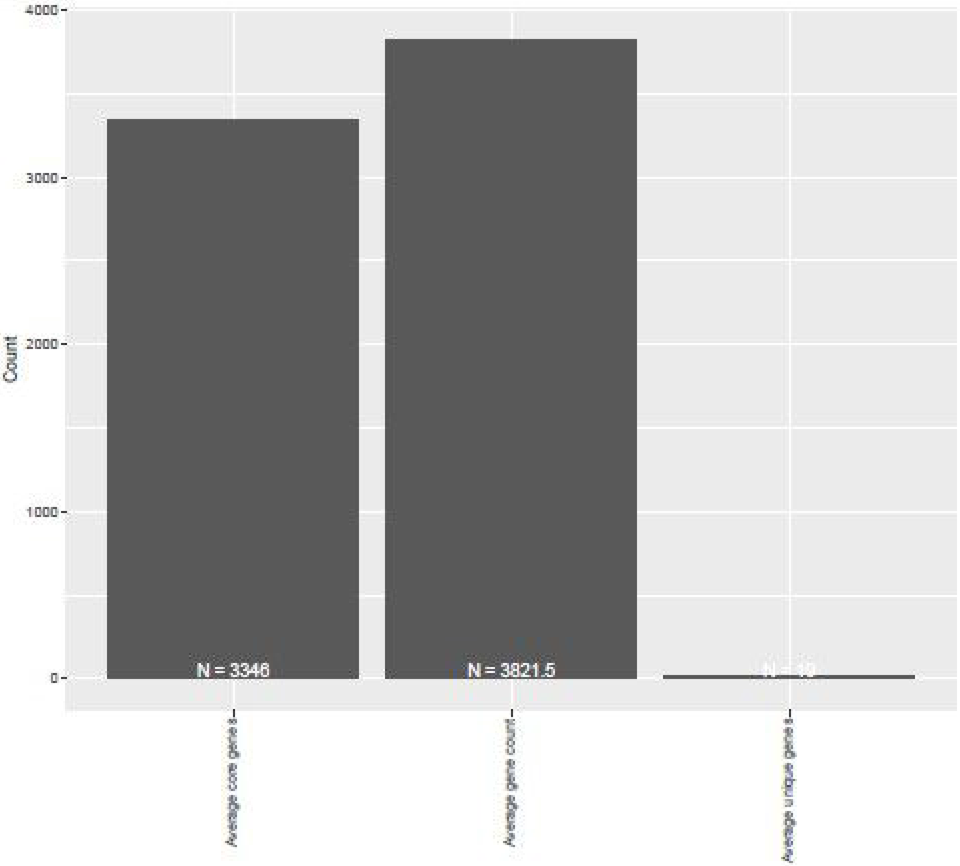

Total number of genomes:
204

Number of analyzed genes:
790409

Total orthologous groups
11759

Total unique genes
3831

Figure S2. Number of Average core genes, average gene count, and average unique genes present in the *B. velezensis* pangenome

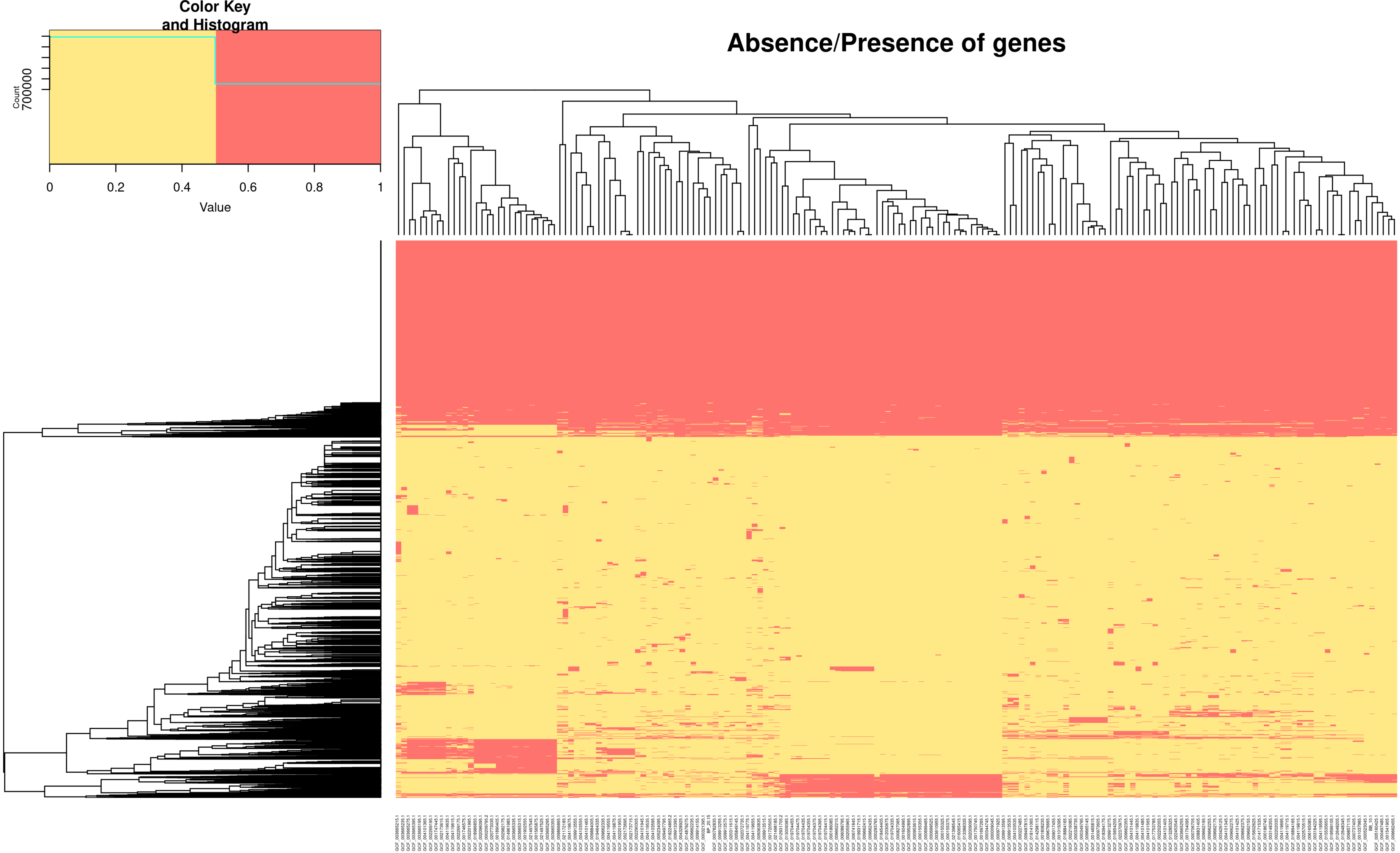

Figure 2. The heatmap (2c) represents the presence and absence of particular genes in BB10.1 and BP20.15 pan-genome. The X-axis denotes the particular specie name. The yellow color indicates gene presence; the red color indicates gene absence. The Y-axis represents individual gene clustering; while the topmost axis represents genomes clustering

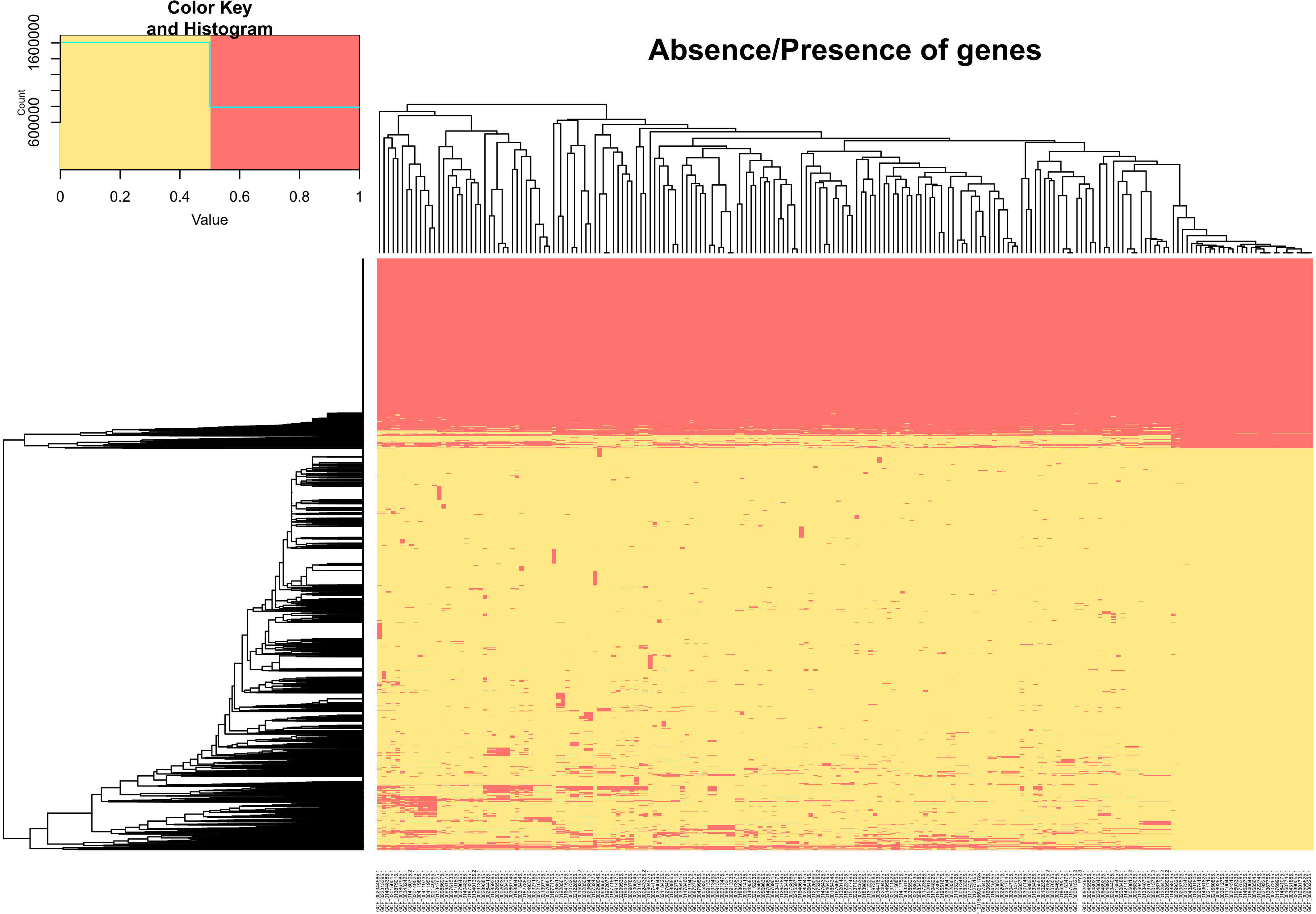

Figure S3.The heatmap represent the presence or absence of a particular genes in PY2.3 pan-genome. The X-axis denotes a particular strain accession number. The yellow color indicates gene presence; the red color indicates gene absence. The Y-axis represents individual gene clustering; while the topmost axis represents the genomes clustering.

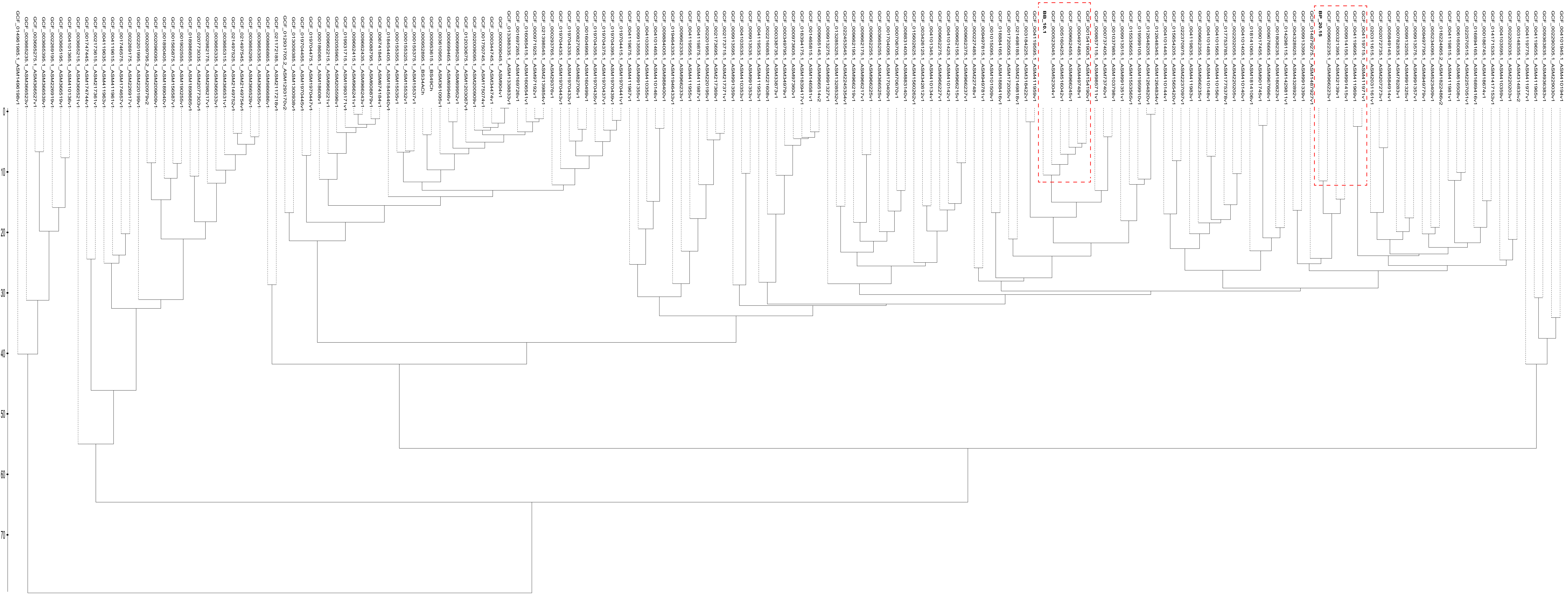

Figure S4a. The phylogenetic inference based on the whole genome variation of isolates BB10.1, BP20.15.

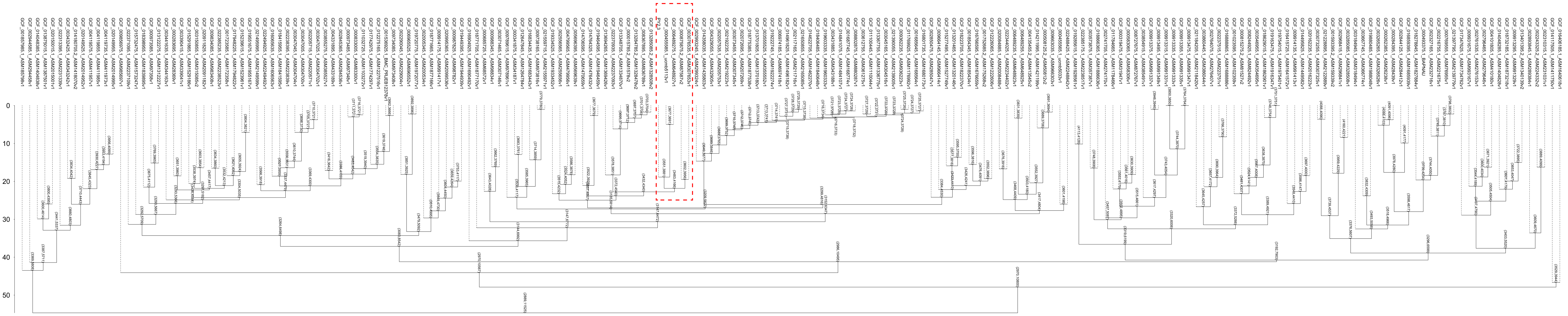

Figure S4b. The phylogenetic inference based on the whole genome variation of isolate PY2.3.

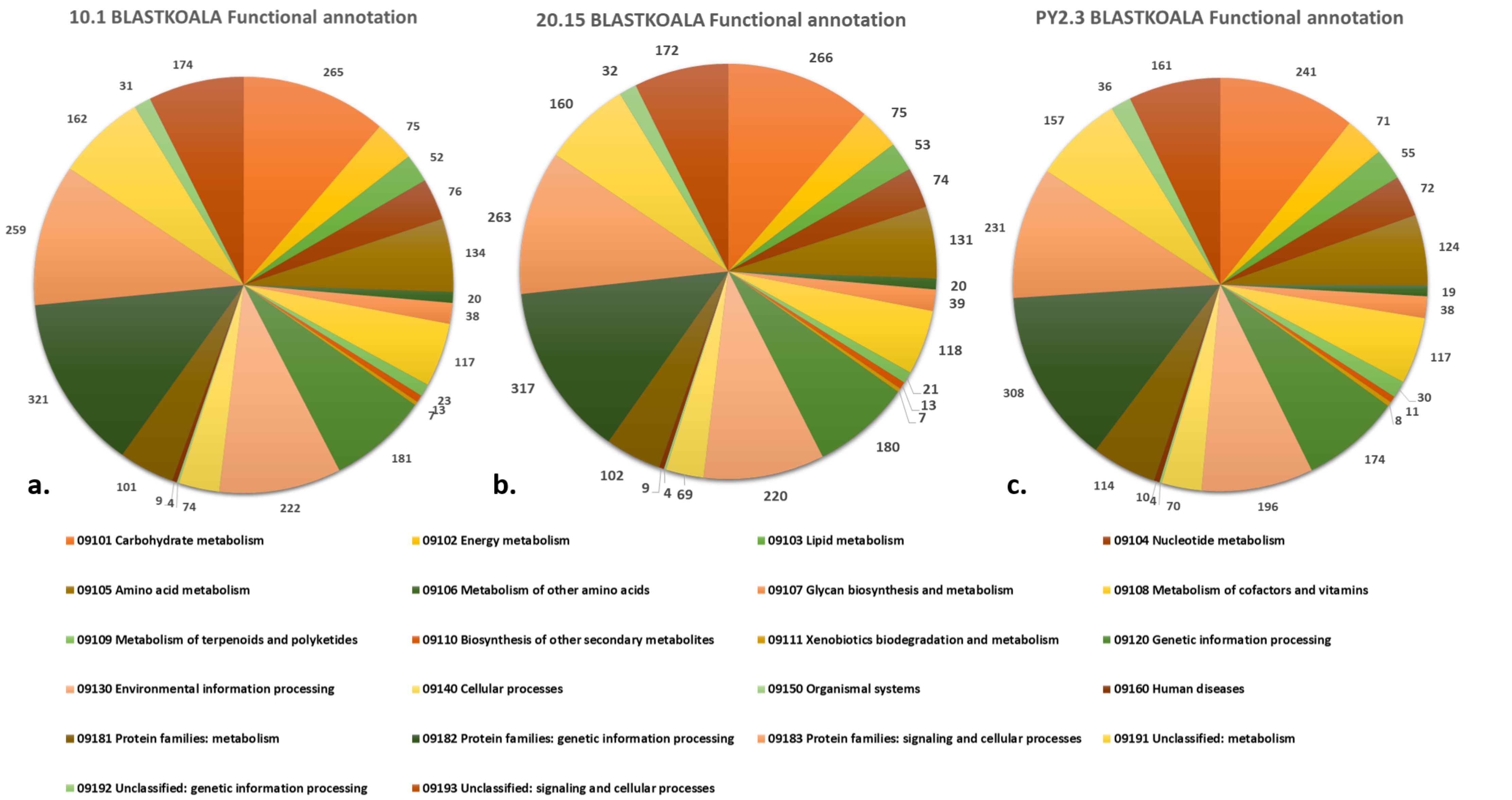

Figure S5 . The KEGG functional annotation by BLASTKOALA and categorization into 22 different functional categories. a) 60% of the annotated sequences from isolate 10.1 b) 61.2% annotated sequences from isolate 20.15 c) 62.6% annotated sequences from isolate PY2.3. The color scheme and code at the bottom represent the specific functional category of annotated genes.
