# Supplementary material for: In Silico Safety Assessment of Bacillus Isolated from Polish Bee Pollen and Bee Bread as Novel Probiotic Candidates
Source: Int J Mol Sci. 2024 Jan 4;25(1):666. doi: 10.3390/ijms25010666 (PMC10780176; doi:10.3390/ijms25010666)
Supplement: Supplementary file 1 [file ijms-25-00666-s001.zip › Supplementary Tables_In Silico Safety Assessment of Bacillus isolated from Polish Bee Pollen and Bee Bread as Novel Probiotic Candidates.pdf]

In Silico Safety Assessment of *Bacillus* isolated from Polish Bee Pollen and Bee Bread as Novel Probiotic Candidates

Ahmer Bin Hafeez[1]; Karolina Pełka[1]; Randy Worobo[2]; Piotr Szweda[1*]

1 - Department of Pharmaceutical Technology and Biochemistry, Faculty of Chemistry, Gdańsk University of Technology, ul. G. Narutowicza 11/12, 80-233 Gdańsk, Poland;

2 - Department of Food Science, Cornell University, Ithaca, NY 14853, USA

Ahmer Bin Hafeez – e-mail: ahmer.bin.hafeez@pg.edu.pl; ORCID: 0000-0002-8712-5898

Karolina Pełka – e-mail: karolina.pelka@pg.edu.pl; ORCID: 0000-0003-2523-8165

Randy Worobo – e-mail: rww8@cornell.edu; ORCID: 0000-0002-5211-3125

Piotr Szweda – email: piotr.szweda@pg.edu.pl; ORCID: 0000-0001-8291-5148[*]

Table S1. Insertion sequence (IS) elements in genomes of the isolates

IS Element	IS Family	Group	Score (bits)	Size (bp)	E. value	Identity %	Inverted repeats (bp)	Direct repeats (bp)	ORF	Function	Origin	Accession number	COMMENTS
**Isolate BB10.1**													
ISBpu1	IS1182		121	1709	3.00E-24	84%	14/15	30	1	Transposase	*B. pumilus*	NC_009848	ISBpu1 is 82% aa similar to ISBcl1
ISBsu1	IS3	IS150	69.9	1289	8.00E-09	95%	32/42	2, 3, 3, 3, 2	3	Transposase	*B. subtilis*	NC_014479	ISBsu1 is 71% aa similar to ISLmo1. The third ORF is the putative ORFAB transposase reconstructed in silico by possible -1 frameshift.
ISBpu1	IS1182		87.8	1709	3.00E-14	85%	14/15	30	1	Transposase	*B. pumilus*	NC_009848	ISBpu1 is 82% aa similar to ISBcl1.
ISBpu1	IS1182		103	1709	2.00E-19	83%	14/15	30	1	Transposase	*B. pumilus*	NC_009848	ISBpu1 is 82% aa similar to ISBcl1.
ISBpu1	IS1182		79.8	1709	2.00E-12	84%	14/15	30	1	Transposase	*B. pumilus*	NC_009848	ISBpu1 is 82% aa similar to ISBcl1.
ISBpu1	IS1182		63.9	1709	1.00E-07	93%	14/15	30	1	Transposase	*B. pumilus*	NC_009848	ISBpu1 is 82% aa similar to ISBcl1.
ISBpu1	IS1182		103	1709	7.00E-20	86%	14/15	30	1	Transposase	*B. pumilus*	NC_009848	ISBpu1 is 82% aa similar to ISBcl1.
ISBspe1	IS1182		61.9	1560	3.00E-07	86%	13/15	23	1	Transposase	*B. pseudofirmus*	NC_013791	ISBspe1 is 79% aa similar to ISOih1.
**Isolate BP20.15**													
ISBsu1	IS3	IS150	69.9	1289	3.00E-09	95%	32/42	2, 3, 3, 3, 2	3	Transposase	*B. subtilis*	NC_014479	ISBsu1 is 71% aa similar to ISLmo1. The third ORF is the putative ORFAB transposase reconstructed in silico by possible -1 frameshift.
ISBpu1	IS3	IS150	103	1709	1.00E-19	83%	14/15	30	1	Transposase	*B. pumilus*	NC_009848	ISBpu1 is 82% aa similar to ISBcl1.
ISBpu1	IS3	IS150	87.7	1709	4.00E-15	85%	14/15	30	1	Transposase	*B. pumilus*	NC_009848	ISBpu1 is 82% aa similar to ISBcl1.
ISBpu1	IS3	IS150	103	1709	6.00E-20	86%	14/15	30	1	Transposase	*B. pumilus*	NC_009848	ISBpu1 is 82% aa similar to ISBcl1.
ISBpu1	IS3	IS150	60	1709	5.00E-07	97%	14/15	30	1	Transposase	*B. pumilus*	NC_009848	ISBpu1 is 82% aa similar to ISBcl1.
ISBpu1	IS3	IS150	89.7	1709	2.00E-16	85%	14/15	30	1	Transposase	*B. pumilus*	NC_009848	ISBpu1 is 82% aa similar to ISBcl1.

ISBpu1	IS3	IS150	81.8	1709	3.00E-16	84%	14/15	30	1	Transposase	*B. pumilus*	NC_009848	ISBpu1 is 82% aa similar to ISBcl1.
**Isolate PY2.3**													
ISBsu1	IS3	IS150	133	1289	4.00E-28	93%	32/42	2, 3, 3, 3, 2	3	Transposase	*B. subtilis*	NC_014479	ISBsu1 is 71% aa similar to ISLmo1. The third ORF is the putative ORFAB transposase reconstructed in silico by possible -1 frameshift
ISBsu1	IS3	IS150	73.8	1289	2.00E-10	93%	32/42	2, 3, 3, 3, 2	3	Transposase	*B. subtilis*	NC_014479	ISBsu1 is 71% aa similar to ISLmo1. The third ORF is the putative ORFAB transposase reconstructed in silico by possible -1 frameshift
ISBsu1	IS3	IS150	73.8	1289	1.00E-10	93%	32/42	2, 3, 3, 3, 2	3	Transposase	*B. subtilis*	NC_014479	ISBsu1 is 71% aa similar to ISLmo1. The third ORF is the putative ORFAB transposase reconstructed in silico by possible -1 frameshift
IS655	IS3		65.9	1221	3.00E-08	93%	9/15	3	3	Transposase	*B. halodurans*	NC_002570	There are 5 copies of IS655 on Bacillus halodurans chromosome
ISBsu1	IS3	IS150	75.8	1289	3.00E-11	93%	32/42	2, 3, 3, 3, 2	3	Transposase	*B. subtilis*	NC_014479	ISBsu1 is 71% aa similar to ISLmo1. The third ORF is the putative ORFAB transposase reconstructed in silico by possible -1 frameshift
ISBsu1	IS3	IS150	107	1289	6.00E-21	97%	32/42	2, 3, 3, 3, 2	3	Transposase	*B. subtilis*	NC_014479	ISBsu1 is 71% aa similar to ISLmo1. The third ORF is the putative ORFAB transposase reconstructed in silico by possible -1 frameshift
ISBsu1	IS3	IS150	107	1289	5.00E-21	97%	32/42	2, 3, 3, 3, 2	3	Transposase	*B. subtilis*	NC_014479	ISBsu1 is 71% aa similar to ISLmo1.

													The third ORF is the putative ORFAB transposase reconstructed in silico by possible -1 frameshift
ISBsu1	IS3	IS150	1402	1289	0.00E+00	97%	32/42	2, 3, 3, 3, 2	3	Transposase	*B. subtilis*	NC_014479	ISBsu1 is 71% aa similar to ISLmo1. The third ORF is the putative ORFAB transposase reconstructed in silico by possible -1 frameshift
ISLmo1	IS3	IS150	50.1	1228	6.00E-06	94%	33/49	0	3	Transposase	*Listeria monocytogenes*	NC_003210	ISLmo1 is 81% (ORFA) and 72% (ORFB) aa similar to ISSau4. The third ORF is a putative ORFAB transposase reconstructed in sillico by -1 frameshift.

Table S2. List of the KEGG pathways related to the biosynthesis of antimicrobial drugs with clinical importance in the genomes of the isolates BB10.1, 20.15, PY2.3.

map_ID	map_Name	Products
	**Metabolism of terpenoids and polyketides**	
map01054	Nonribosomal peptide structures	Lichenysin, fengycin, and surfactin
map01055	Biosynthesis of vancomycin group antibiotics	Vancomycin
map00900	Terpenoid backbone biosynthesis	Essential oil monoterpenes and linalyl acetate, sesquiterpenes, diterpenes, triterpenes, and carotenoids, phytol and sterols.
map00906	Carotenoid biosynthesis	beta-carotene
map00981	Insect hormone biosynthesis	Juvenile hormone
map00908	Zeatin biosynthesis	Zeatin

map00903	Limonene and pinene degradation	Multiple products
map00281	Geraniol degradation	Geranoil byproducts
map01051	Biosynthesis of ansamycins	Rifamycin
map00523	Polyketide sugar unit biosynthesis	Multiple products
map01053	Biosynthesis of siderophore group nonribosomal peptides	Bacillibactin
**Biosynthesis of other secondary metabolites**		
map00940	Phenylpropanoid biosynthesis	Monolignol
map00950	Isoquinoline alkaloid biosynthesis	Naringenin
map00960	Tropane, piperidine and pyridine alkaloid biosynthesis	Reticuline, morphine, sanguinarine, noscapine
map00232	Caffeine metabolism	Xanthine
map00966	Glucosinolate biosynthesis	Glucobrassicin
map00311	Penicillin and cephalosporin biosynthesis	Penicillin, cephamycin C
map00332	Carbapenem biosynthesis	Carbapenem
map00261	Monobactam biosynthesis	nocardicin A
map00521	Streptomycin biosynthesis	Streptomycin (INN)
map00524	Neomycin, kanamycin and gentamicin biosynthesis	Tobramycin (JP18/USP), Apramycin (USAN/INN), Framycetin (INN), Paromomycin (INN), Bekanamycin (INN), Micronomicin (DCF), Ribostamycin (INN)
map00525	Acarbose and validamycin biosynthesis	validamycin A
map00401	Novobiocin biosynthesis	Coumermycin (USAN)
map00405	Phenazine biosynthesis	pyocyanine
map00333	Prodigiosin biosynthesis	Prodigiosin, undecylprodigiosin
map00998	Biosynthesis of various antibiotics	Kanosamine, Aurachin, Bacilysin, Puromycin, Dapdiamides, Fosfomycin, Cremeomycin, Pentalenolactone, Terpentecin, Roseoflavin, Cycloserine
map00999	Biosynthesis of various plant secondary metabolites	Crocin, Cannabidiol, Mugineic acid, Pentagalloylglucose, Benzoxazinoid, Gramine, Coumarin, Furanocoumarin, Hordatine, Podophyllotoxin

Table S3. CARD database results for the antibiotic resistance genes in the genome of the isolates.

Contig	Start	Stop	Orientation	Cut_Off	Pass_Bitscore	Best_Hit_Bitscore	Best_Hit_ARO	Best_Identities	ARO	Drug Class	Resistance Mechanism	AMR Gene Family
**Isolate BB10.1**												
lcl|NODE1_51	51517	51951	-	Strict	150	183	FosBx1	62.04	3007372	phosphonic acid antibiotic	antibiotic inactivation	fosfomycin thiol transferase
lcl|NODE1_74	69489	70673	+	Strict	175	213	vanT gene in vanG cluster	34.05	3002972	glycopeptide antibiotic	antibiotic target alteration	glycopeptide resistance gene cluster; vanT
lcl|NODE1_162	145443	145760	+	Strict	75	97.8	qacJ	46.46	3007014	disinfecting agents and antiseptics	antibiotic efflux	small multidrug resistance (SMR) antibiotic efflux pump
lcl|NODE1_163	145774	146127	+	Strict	75	94.4	qacJ	44.9	3007014	disinfecting agents and antiseptics	antibiotic efflux	small multidrug resistance (SMR) antibiotic efflux pump
lcl|NODE1_595	633647	633964	-	Perfect	180	205.7	ykkD	100	3003064	aminoglycoside antibiotic; tetracycline antibiotic; phenicol antibiotic	antibiotic efflux	small multidrug resistance (SMR) antibiotic efflux pump
lcl|NODE1_596	633964	634302	-	Perfect	180	220.7	ykkC	100	3003063	aminoglycoside antibiotic; tetracycline antibiotic; phenicol antibiotic	antibiotic efflux	small multidrug resistance (SMR) antibiotic efflux pump
lcl|NODE2_42	46909	47820	-	Strict	50	119	vanW gene in vanI cluster	37.87	3003724	glycopeptide antibiotic	antibiotic target alteration	vanW; glycopeptide resistance gene cluster
lcl|NODE2_50	53514	54434	+	Strict	350	405.2	BcI	65.7	3002877	cephalosporin; penem	antibiotic inactivation	class A Bacillus cereus Bc beta-lactamase
lcl|NODE2_144	139548	140369	-	Strict	50	112.1	vanY gene in vanG cluster	35.37	3002959	glycopeptide antibiotic	antibiotic target alteration	vanY; glycopeptide resistance gene cluster
lcl|NODE2_662	587547	588749	+	Perfect	750	740.7	blt	100	3003006	fluoroquinolone antibiotic; disinfecting agents and antiseptics	antibiotic efflux	major facilitator superfamily (MFS) antibiotic efflux pump
lcl|NODE2_682	606252	607106	-	Perfect	500	596.3	aadK	100	3002627	aminoglycoside antibiotic	antibiotic inactivation	ANT(6)
lcl|NODE3_268	270249	270608	-	Strict	75	86.3	qacG	42.16	3007015	disinfecting agents and antiseptics	antibiotic efflux	small multidrug resistance (SMR)

												antibiotic efflux pump
lcl|NODE4_33	27386	29956	-	Perfect	1650	1635.2	Bacillus subtilis mprF	100	3003324	peptide antibiotic	antibiotic target alteration	defensin resistant mprF
lcl|NODE6_123	121545	122714	+	Strict	175	186.8	vanT gene in vanG cluster	32.05	3002972	glycopeptide antibiotic	antibiotic target alteration	glycopeptide resistance gene cluster; vanT
lcl|NODE6_205	188399	190042	-	Perfect	1000	1063.1	vmlR	100	3004476	lincosamide antibiotic; streptogramin antibiotic; streptogramin B antibiotic	antibiotic target protection	Miscellaneous ABC-F subfamily ATP-binding cassette ribosomal protection proteins
lcl|NODE7_95	99651	100571	+	Perfect	500	631.7	mphK	100	3004541	macrolide antibiotic	antibiotic inactivation	macrolide phosphotransferase (MPH)
lcl|NODE7_157	162969	163562	-	Perfect	375	401.4	tmrB	100	3003059	nucleoside antibiotic	reduced permeability to antibiotic	tunicamycin resistance protein
lcl|NODE8_97	90607	91983	-	Strict	450	682.9	tet(45)	75.82	3003196	tetracycline antibiotic	antibiotic efflux	major facilitator superfamily (MFS) antibiotic efflux pump
**BP20.15**												
lcl|1_439	384141	384962	+	Strict	50	111.7	vanY gene in vanM cluster	34.55	3002961	glycopeptide antibiotic	antibiotic target alteration	vanY; glycopeptide resistance gene cluster
lcl|1_525	468553	469473	-	Strict	350	408.7	BcI	66.02	3002877	cephalosporin; penem	antibiotic inactivation	class A Bacillus cereus Bc beta-lactamase
lcl|1_533	475198	476109	+	Strict	50	119	vanW gene in vanI cluster	37.87	3003724	glycopeptide antibiotic	antibiotic target alteration	vanW; glycopeptide resistance gene cluster
lcl|2_311	313264	314118	+	Strict	500	585.9	aadK	98.24	3002627	aminoglycoside antibiotic	antibiotic inactivation	ANT(6)
lcl|4_73	69148	70332	+	Strict	175	212.6	vanT gene in vanG cluster	34.05	3002972	glycopeptide antibiotic	antibiotic target alteration	glycopeptide resistance gene cluster; vanT
lcl|4_110	103150	103467	+	Strict	75	97.8	qacJ	46.46	3007014	disinfecting agents and antiseptics	antibiotic efflux	small multidrug resistance (SMR) antibiotic efflux pump
lcl|4_111	103481	103834	+	Strict	75	94.4	qacJ	44.9	3007014	disinfecting agents and antiseptics	antibiotic efflux	small multidrug resistance (SMR) antibiotic efflux pump
lcl|5_215	202890	203207	-	Strict	180	203	ykkD	99.05	3003064	aminoglycoside antibiotic;	antibiotic efflux	small multidrug resistance (SMR)

										tetracycline antibiotic; phenicol antibiotic		antibiotic efflux pump
lcl|5_216	203207	203545	-	Perfect	180	220.7	ykkC	100	3003063	aminoglycoside antibiotic; tetracycline antibiotic; phenicol antibiotic	antibiotic efflux	small multidrug resistance (SMR) antibiotic efflux pump
lcl|7_39	46816	47409	+	Strict	375	395.6	tmrB	97.97	3003059	nucleoside antibiotic	reduced permeability to antibiotic	tunicamycin resistance protein
lcl|7_101	109799	110719	-	Strict	500	624	mphK	98.69	3004541	macrolide antibiotic	antibiotic inactivation	macrolide phosphotransferase (MPH)
lcl|14_72	70902	72278	-	Strict	450	677.2	tet(45)	74.95	3003196	tetracycline antibiotic	antibiotic efflux	major facilitator superfamily (MFS) antibiotic efflux pump
lcl|15_4	2364	3533	+	Strict	175	187.2	vanT gene in vanG cluster	32.05	3002972	glycopeptide antibiotic	antibiotic target alteration	glycopeptide resistance gene cluster; vanT
lcl|15_87	73116	74759	-	Strict	1000	1042	vmlR	98.35	3004476	lincosamide antibiotic; streptogramin antibiotic; streptogramin B antibiotic	antibiotic target protection	Miscellaneous ABC-F subfamily ATP-binding cassette ribosomal protection proteins
lcl|20_21	16958	17317	+	Strict	75	86.3	qacG	42.16	3007015	disinfecting agents and antiseptics	antibiotic efflux	small multidrug resistance (SMR) antibiotic efflux pump
**Isolate PY2.3**												
lcl|1_307	332022	332936	-	Strict	350	391.3	BcI	63.07	3002877	cephalosporin; penem	antibiotic inactivation	class A *B. cereus* Bc beta-lactamase
lcl|2_512	505358	506539	-	Strict	175	202.2	vanT gene in vanG cluster	33.96	3002972	glycopeptide antibiotic	antibiotic target alteration	glycopeptide resistance gene cluster; vanT
lcl|3_126	161457	161771	+	Strict	75	82.8	qacJ	37.37	3007014	disinfecting agents and antiseptics	antibiotic efflux	small multidrug resistance (SMR) antibiotic efflux pump
lcl|3_127	161789	162142	+	Strict	75	93.2	qacJ	44.9	3007014	disinfecting agents and antiseptics	antibiotic efflux	small multidrug resistance (SMR) antibiotic efflux pump

lcl|10_26	25546	25911	-	Strict	75	79.7	qacG	42.45	3007015	disinfecting agents and antiseptics	antibiotic efflux	small multidrug resistance (SMR) antibiotic efflux pump
lcl|10_53	49262	50431	+	Strict	175	192.2	vanT gene in vanG cluster	34.25	3002972	glycopeptide antibiotic	antibiotic target alteration	glycopeptide resistance gene cluster; vanT
lcl|10_138	115803	116852	+	Strict	600	701	clbA	99.43	3002814	lincosamide antibiotic; streptogramin antibiotic; streptogramin A antibiotic; oxazolidinone antibiotic; phenicol antibiotic; pleuromutilin antibiotic	antibiotic target alteration	Cfr 23S ribosomal RNA methyltransferase
lcl|10_191	164755	165069	-	Strict	75	75.1	qacG	37.25	3007015	disinfecting agents and antiseptics	antibiotic efflux	small multidrug resistance (SMR) antibiotic efflux pump
lcl|12_48	36737	37555	+	Strict	50	105.9	vanY gene in vanB cluster	34.87	3002956	glycopeptide antibiotic	antibiotic target alteration	vanY; glycopeptide resistance gene cluster

Table S4. VirulenceFinder database results for virulence genes in the isolates BB 10.1, BP 20.15 and PY2.3 genomes

**VFclass**	**Virulence factors**	**Related genes**	**Isolate BB10.1**	***B.subtilis* subsp. subtilis str. 168**
			**draft**	**chromosome (NC_000964)**
Adherence	BslA	bslA	-	-
Enzyme	Sphingomyelinase (SMase)	sph	-	-
	Phosphatidylinositol-specific phospholipase C (PI-PLC)	piplc	-	-
	Phosphatidylcholine-preferring phospholipase C (PC-PLC)	plcA	-	-
	Immune inhibitor A metalloproteinase	Undetermined	-	-
		inhA	-	-
Immune evasion	Polysaccharide capsule	Undetermined	-	-
	Polyglutamic acid capsule	capA	pgaptmp_002865	BSU35880
		capB	pgaptmp_002867	BSU35900
		capC	pgaptmp_002866	BSU35890
		capD	pgaptmp_001187	BSU18410
		capE	-	-
	Hyaluronic acid (HA) capsule	hasA	-	-
		hasB	-	-
		hasC	-	-
	B. cereus exo-polysaccharide (BPS)	bpsA	-	-
		bpsB	-	-
		bpsC	-	-
		bpsD	-	-
		bpsE	-	-
		bpsF	-	-

		bpsG	-	-
		bpsH	-	-
		bpsX	-	-
Iron acquisition	Petrobactin	asbA	-	-
		asbB	-	-
		asbC	-	-
		asbD	-	-
		asbE	-	-
		asbF	-	-
	IlsA	ilsA	-	-
	Hal	hal	-	-
	Bacillibactin	dhbA	pgaptmp_002461	BSU32000
		dhbB	pgaptmp_002458	BSU31970
		dhbC	pgaptmp_002460	BSU31990
		dhbE	pgaptmp_002459	BSU31980
		dhbF	pgaptmp_002457	BSU31960
Regulation	PlcR-PapR quorum sensing	papR	-	-
		plcR	-	-
	PagR-XO2	pagR-XO2	-	-
	PagR-XO1	pagR-XO1	-	-
	AtxA	atxA	-	-
	AcpAB	acpA	-	-
		acpB	-	-
Secretion system	Type VII secretion system	-	-	-
		-	-	-
		essC	-	-

		esxB	-	-
		esxL	-	-
Toxin	Non-hemolytic enterotoxin (Nhe)	nheA	-	-
		nheB	-	-
		nheC	-	-
	Insecticidal crystalline toxins	cry	-	-
		cyt	-	-
		vip	-	-
	Hemolytic enterotoxin HBL	hblA	-	-
		hblC	-	-
		hblD	-	-
	Hemolysin III homolog	Undetermined	-	-
	Hemolysin III	hlyIII	pgaptmp_001360	BSU21790
	Hemolysin II	hlyII	-	-
	Cytotoxin K (Hemolysin IV)	cytK	-	-
	Certhrax	cer	-	-
	Cereulide	cesA	-	-
		cesB	-	-
		cesC	-	-
		cesD	-	-
		cesH	-	-
		cesP	-	-
		cesT	-	-
	Anthrolysin O/Cereolysin O/Hemolysin I	alo	-	-
	Anthrax toxin	cya	-	-
		lef	-	-

		pagA	-	-

VFclass	Virulence factors	Related genes	BP20.15 (Prediction)	B.subtilis subsp. subtilis str. 168
			**draft (draft)**	**chromosome (NC_000964)**
Adherence	Flagella(Pseudomonas)	fliP	pgaptmp_000987	-
	Fibronectin-binding protein(Listeria)	fbpA	pgaptmp_000915	-
	D-alanine-polyphosphoribitol ligase(Listeria)	dltA	pgaptmp_000589	-
	BslA	bslA	-	-
Enzyme	Serine-threonine phosphatase(Listeria)	stp	pgaptmp_000927	-
	Sphingomyelinase (SMase)	sph	-	-
	Phosphatidylinositol-specific phospholipase C (PI-PLC)	piplc	-	-
	Phosphatidylcholine-preferring phospholipase C (PC-PLC)	plcA	-	-
	Immune inhibitor A metalloproteinase	Undetermined	-	-
		inhA	-	-
Immune evasion	Polysaccharide capsule	Undetermined	pgaptmp_000779; pgaptmp_000790; pgaptmp_000791; pgaptmp_001361; pgaptmp_002843; pgaptmp_002923; pgaptmp_003428; pgaptmp_003439; pgaptmp_003458; pgaptmp_003503; pgaptmp_003504; pgaptmp_003505; pgaptmp_003506	-
	Polyglutamic acid capsule	capA	pgaptmp_003467	BSU35880

		capB	pgaptmp_003469	BSU35900
		capC	pgaptmp_003468	BSU35890
		capD	pgaptmp_002807	BSU18410
		capE	-	-
	Hyaluronic acid (HA) capsule	hasA	-	-
		hasB	-	-
		hasC	-	-
	B. cereus exo-polysaccharide (BPS)	bpsA	-	-
		bpsB	-	-
		bpsC	-	-
		bpsD	-	-
		bpsE	-	-
		bpsF	-	-
		bpsG	-	-
		bpsH	-	-
		bpsX	-	-
Iron acquisition	Petrobactin	asbA	-	-
		asbB	-	-
		asbC	-	-
		asbD	-	-
		asbE	-	-
		asbF	-	-
	IlsA	ilsA	-	-
	Hal	hal	-	-
	Bacillibactin	dhbA	pgaptmp_003945	BSU32000
		dhbB	pgaptmp_003948	BSU31970

		dhbC	pgaptmp_003946	BSU31990
		dhbE	pgaptmp_003947	BSU31980
		dhbF	pgaptmp_003949	BSU31960
Regulation	CheA/CheY(Listeria)	cheY	pgaptmp_000985; pgaptmp_002949	-
	Carbon storage regulator A(Legionella)	csrA	pgaptmp_002493	-
	PlcR-PapR quorum sensing	papR	-	-
		plcR	-	-
	PagR-XO2	pagR-XO2	-	-
	PagR-XO1	pagR-XO1	-	-
	AtxA	atxA	-	-
	AcpAB	acpA	-	-
		acpB	-	-
Secretion system	Type III secretion system(Chlamydia)	cdsN	pgaptmp_000975	-
	Type VII secretion system	-	-	-
		-	-	-
		essC	-	-
		esxB	-	-
		esxL	-	-
Toxin	Non-hemolytic enterotoxin (Nhe)	nheA	-	-
		nheB	-	-
		nheC	-	-
	Insecticidal crystalline toxins	cry	-	-
		cyt	-	-
		vip	-	-
	Hemolytic enterotoxin HBL	hblA	-	-

		hblC	-	-
		hblD	-	-
	Hemolysin III homolog	Undetermined	-	-
	Hemolysin III	hlyIII	pgaptmp_000416	BSU21790
	Hemolysin II	hlyII	-	-
	Cytotoxin K (Hemolysin IV)	cytK	-	-
	Certhrax	cer	-	-
	Cereulide	cesA	-	-
		cesB	-	-
		cesC	-	-
		cesD	-	-
		cesH	-	-
		cesP	-	-
		cesT	-	-
	Anthrolysin O/Cereolysin O/Hemolysin I	alo	-	-
	Anthrax toxin	cya	-	-
		lef	-	-
		pagA	-	-
Acid resistance	Urease(Helicobacter)	ureB	pgaptmp_003552	-
Antiphagocytosis	Capsule(Enterococcus)		pgaptmp_003099	-
	Capsular polysaccharide(Vibrio)	wcaJ	pgaptmp_000781	-
Copper uptake	Copper exporter(Mycobacterium)	ctpV	pgaptmp_002083	-
Iron uptake	Periplasmic binding protein-dependent ABC transport systems(Vibrio)	vctC	pgaptmp_001038	-
	Mycobactin(Mycobacterium)	mbtH	pgaptmp_003950	-
	Achromobactin biosynthesis and transport(Pseudomonas)	cbrD	pgaptmp_002584	-

Peptidoglycan modification	OatA(Listeria)	oatA	pgaptmp_001971	-
Stress adaptation	Catalase(Neisseria)	katA	pgaptmp_004191	-
Surface protein anchoring	Lipoprotein-specific signal peptidase II(Listeria)	lspA	pgaptmp_000896	-
	Lipoprotein diacylglyceryl transferase(Listeria)	lgt	pgaptmp_000857	-

**VFclass**	**Virulence factors**	**Related genes**	**PY2.3 (Prediction)**	**B.subtilis subsp. subtilis str. 168**
			**draft (draft)**	**chromosome (NC_000964)**
Adherence	BslA	bslA	-	-
Enzyme	Sphingomyelinase (SMase)	sph	-	-
	Phosphatidylinositol-specific phospholipase C (PI-PLC)	piplc	-	-
	Phosphatidylcholine-preferring phospholipase C (PC-PLC)	plcA	-	-
	Immune inhibitor A metalloproteinase	Undetermined	-	-
		inhA	-	-
Immune evasion	Polysaccharide capsule	Undetermined	pgaptmp_000306; pgaptmp_002467; pgaptmp_002468; pgaptmp_002711	-
	Polyglutamic acid capsule	capA	pgaptmp_002428	BSU35880
		capB	pgaptmp_002430	BSU35900
		capC	pgaptmp_002429	BSU35890
		capD	pgaptmp_001205	BSU18410
		capE	-	-
	Hyaluronic acid (HA) capsule	hasA	-	-
		hasB	-	-
		hasC	-	-
	B. cereus exo-polysaccharide (BPS)	bpsA	-	-

		bpsB	-	-
		bpsC	-	-
		bpsD	-	-
		bpsE	-	-
		bpsF	-	-
		bpsG	-	-
		bpsH	-	-
		bpsX	-	-
Iron acquisition	Petrobactin	asbA	-	-
		asbB	-	-
		asbC	-	-
		asbD	-	-
		asbE	-	-
		asbF	-	-
	IlsA	ilsA	-	-
	Hal	hal	-	-
	Bacillibactin	dhbA	pgaptmp_003354	BSU32000
		dhbB	pgaptmp_003351	BSU31970
		dhbC	pgaptmp_003353	BSU31990
		dhbE	pgaptmp_003352	BSU31980
		dhbF	pgaptmp_003350	BSU31960
Regulation	PlcR-PapR quorum sensing	papR	-	-
		plcR	-	-
	PagR-XO2	pagR-XO2	-	-
	PagR-XO1	pagR-XO1	-	-
	AtxA	atxA	-	-

	AcpAB	acpA	-	-
		acpB	-	-
Secretion system	Type VII secretion system	-	-	-
		-	-	-
		essC	-	-
		esxB	-	-
		esxL	-	-
Toxin	Non-hemolytic enterotoxin (Nhe)	nheA	-	-
		nheB	-	-
		nheC	-	-
	Insecticidal crystalline toxins	cry	-	-
		cyt	-	-
		vip	-	-
	Hemolytic enterotoxin HBL	hblA	-	-
		hblC	-	-
		hblD	-	-
	Hemolysin III homolog	Undetermined	-	-
	Hemolysin III	hlyIII	pgaptmp_001084	BSU21790
	Hemolysin II	hlyII	-	-
	Cytotoxin K (Hemolysin IV)	cytK	-	-
	Certhrax	cer	-	-
	Cereulide	cesA	-	-
		cesB	-	-
		cesC	-	-
		cesD	-	-
		cesH	-	-

		cesP	-	-
		cesT	-	-
	Anthrolysin O/Cereolysin O/Hemolysin I	alo	-	-
	Anthrax toxin	cya	-	-
		lef	-	-
		pagA	-	-
Cell surface components	GPL locus(Mycobacterium)	mbtH	pgaptmp_003349	-

Table S5a. List of identified secondary metabolite cluster of isolate BP20.15 using strictness 'strict'

Region	Type	From	To	Most similar known cluster	Similarity
Region 1.1	T3PKS	315,873	356,970	1-carbapen-2-em-3-carboxylic acid; Other	---
Region 1.2	terpene	405,447	427,345		
Region 4.1	NRPS, betalactone	1	27,594	Fengycin; NRP	80%
Region 4.2	NRPS, transAT-PKS, T3PKS	99,515	204,780	Bacillaene; Polyketide+NRP	100%
Region 6.1	Sactipeptide	196,374	217,985	subtilosin A; RiPP:Thiopeptide	100%
Region 7.1	NRPS	1	28,058	Surfactin; NRP:Lipopeptide	39%
Region 7.2	Sactipeptide, ranthipeptide	161,078	184,031	sporulation killing factor; RiPP:Head-to-tailcyclized peptide	100%
Region 8.1	NRP-metallophore, NRPS	77,003	128,780	Bacillibactin; NRP	100%
Region 11.1	CDPS	128,855	144,076	Pulcherriminic acid; Other	100%
Region 13.1	NRPS	1	25,377	Surfactin; NRP:Lipopeptide	43%
Region 16.1	Other	1	35,408	Bacilysin; Other	100%

Region 19.1	terpene	26,099	46,902		
Region 36.1	NRPS	1	13,319	Plipastatin; NRP	38%
Region 39.1	NRPS	1	10,270	Plipastatin; NRP	23%
Region 40.1	NRPS	1	9,571	Surfactin; NRP:Lipopeptide	8%

Table S5b. List of identified secondary metabolite cluster of isolate PY2.3 using strictness 'strict'

Region 1.1	transAT-PKS	27,584	115,813	macrolactin H; Polyketide	100%
Region 1.2	terpene	463,273	484,013		
Region 2.1	transAT-PKS	530,805	564,859	Difficidin; Polyketide	46%
Region 3.1	betalactone,NRPS,transAT-PKS	1	87,040	Fengycin; NRP	73%
Region 3.2	transAT-PKS, NRPS, T3PKS	156,317	258,960	Bacillaene Polyketide+NRP	100%
Region 4.1	Other	175,848	217,266	Bacilysin; Other	100%
Region 5.1	NRPS	55,796	121,203	Surfactin NRP:Lipopeptide	91%
Region 7.1	NRP-metallophore, NRPS	73,402	125,137	Bacillibactin NRP	100%
Region 9.1	T3PKS	1	30,402		
Region 12.1	terpene	61,208	83,091		
Region 13.1	NRPS	1	39,930		
Region 18.1	NRPS	1	12,626	Plipastatin; NRP	30%
Region 19.1	NRPS	1	9,332	Fengycin; NRP	13%
